# Polyamine–Oligonucleotide Conjugates: 2′-*O*Me-Triazole-Linked 1,4,7,10-Tetraazacyclododecane and Intercalating Dyes and Their Effect on the Thermal Stability of DNA Duplexes

**DOI:** 10.3390/pharmaceutics14010066

**Published:** 2021-12-28

**Authors:** Mateusz D. Tomczyk, Mariusz Zalewski, Per T. Jørgensen, Jesper Wengel, Krzysztof Walczak

**Affiliations:** 1 Department of Organic Chemistry, Bioorganic Chemistry and Biotechnology, Silesian University of Technology, Krzywoustego 4, 44-100 Gliwice, Poland; mateusz.d.tomczyk@polsl.pl; 2 Department of Chemical Organic Technology and Petrochemistry, Silesian University of Technology, Krzywoustego 4, 44-100 Gliwice, Poland; mariusz.zalewski@polsl.pl; 3 BioNEC Center, Department of Physics, Chemistry and Pharmacy, University of Southern Denmark, Campusvej 55, 5230 Odense M, Denmark; ptj@sdu.dk (P.T.J.); jwe@sdu.dk (J.W.)

**Keywords:** oligonucleotide conjugates, duplex stability, thioxanthone, 1,8-napthalimide, cyclen

## Abstract

Oligonucleotides with the sequences 5′-GTG AU^P^A TGC, 5′-GCA TAU^P^ CAC and 5′-GU^P^G ATA U^P^GC, where U^P^ is 2′-*O*-propargyl uridine, were subjected to post-synthetic Cu(I)-catalyzed azide–alkyne cycloaddition to attach 1,4,7,10-tetraazacyclododecane (cyclen) and two well-known DNA intercalating dyes: thioxanthone and 1,8-naphthalimide. We propose a convenient cyclen protection–deprotection strategy that allows efficient separation of the resulting polyamine–oligonucleotide conjugates from the starting materials by RP-HPLC to obtain high-purity products. In this paper, we present hitherto unknown macrocyclic polyamine–oligonucleotide conjugates and their hybridization properties reflected in the thermal stability of thirty-two DNA duplexes containing combinations of labeled strands, their unmodified complementary strands, and strands with single base pair mismatches. Circular dichroism measurements showed that the B-conformation is retained for all dsDNAs consisting of unmodified and modified oligonucleotides. An additive and destabilizing effect of cyclen moieties attached to dsDNAs was observed. *T*_m_ measurements indicate that placing the hydrophobic dye opposite to the cyclen moiety can reduce its destabilizing effect and increase the thermal stability of the duplex. Interestingly, the cyclen-modified U showed significant selectivity for TT mismatch, which resulted in stabilization of the duplex. We conclude the paper with a brief review and discussion in which we compare our results with several examples of oligonucleotides labeled with polyamines at internal strand positions known in the literature.

## 1. Introduction

Macrocyclic polyamines and their transition metal complexes are attracting increasing interest due to their clinical potential in cancer and virus treatment and in magnetic resonance imaging. Chemical modifications involving covalent attachment of polyamines to oligonucleotides (ON) create zwitterionic functional groups that can significantly improve their biological and biophysical properties, such as target affinity and cell penetration, in a manner similar to polyamine transfection agents. The introduction of such modifications was carried out using several different strand positions, including the 3′ and 5′-positions of the phosphate backbone, the 2′ and 4′-positions on the ribose ring, and within the nucleobase itself [1,2]. In contrast to the 3′ and 5′-positions, the stability of both oligonucleotides and duplexes is more sensitive to modifications of the ribose ring structure and conformation, although it is the 2′-position of the ribose ring that is particularly suitable for the covalent attachment of large molecules, such as polyamines, with minimal disruption of the base-paring potential. There are many examples of polyamine–oligonucleotide conjugates, but in most studies, polyamines are linear, while examples of macrocyclic polyamines that can form stable complexes with transition metals are rarer, and information on their effect on duplex stability is often lacking. Dubey et al. showed that cyclen-based transition metal complexes attached to the 5′-position of an oligo(dT) are able to hydrolyze the target oligo(dA) more efficiently; however, the effect of the cyclen moiety on the thermal stability of the duplex has not been described [3]. Steward et al. demonstrated a four-arm, lattice-bearing, single-stranded DNA bound to the central Ni(II)–cyclen complex, which improves self-assembly at the supramolecular level, but its effect on duplexes is also unknown [4]. On the other hand, it is known that macrocyclic polyamines such as 1,4,7,10- tetraazacyclododecane (cyclen) are potential artificial nucleases, and their derivatives can cleave double-stranded DNA (dsDNA), even without metal ions, through hydrolysis or oxidative cleavage [5,6,7,8,9]. Thus, covalent attachment of a macrocyclic amine to ssDNA may provide new and useful models for studying the function and in vitro use of artificial nucleic acid-based nucleases.

The second type of modification that we introduced, intercalating dyes, have a considerable position in the chemistry of nucleic acids [10]. These planar and aromatic molecules can intercalate between the nucleobases of dsDNA changing its topology but can also be explored as fluorescent probes for in vitro applications. Typically, oligonucleotide-based probes consist of covalently attached fluorescent dyes, including perylene [11], pyrene and phenanthroline [12,13,14,15], or fluorescein [16], which are known to exhibit high fluorescence and can interact noncovalently with dsDNA, e.g., by intercalation or groove-binding, leading to its stabilization. We have previously shown that covalent attachment of a carbazole moiety to the 5′-end of a 9-mer sequence increases the thermal stability of the resulting 9-mer/15-mer dsDNA by +4.2 °C [17]. To date, the effect of the combined attachment of both molecules, an intercalator and cyclen, to double-stranded oligonucleotides on their thermal stability has not been investigated. The knowledge of the stabilizing (or destabilizing) effect will be helpful in the preparation of cyclen-containing oligonucleotides with tailored stability of the resulting hybridized duplexes. Telser et al. prepared several dsDNAs with covalently attached labels, e.g., anthraquinone or pyrene, placed at the internal positions of both strands and showed that both label–duplex and label–label interactions affect the thermal stability of the resulting duplexes [18,19]. Following the above studies, we also examined the mutual influence of the introduced modifications on the stability of duplexes.

Herein, we present a preliminary study of a new methodology for the covalent attachment of cyclen moieties to oligonucleotides and the assessment of their effect on the thermal stability of the resulting DNA duplexes. For this purpose, we developed a new procedure for introducing *N*-TFA-protected cyclen via a 2′-*O*Me-triazole linkage, followed by purification and deprotection of the resulting conjugate to obtain a high-purity product that is well separated from the initial oligonucleotide. We were also interested in the mutual influence of the different labels placed on opposite positions of complementary strands on their stabilizing properties, which turn out to be of significant importance in the case of cyclen groups. In summary, we tested seven labeled oligonucleotides on examples of thirty-two dsDNA combinations formed between the labeled strands, their unmodified complementary strands, and strands with a single base pair mismatch.

## 2. Materials and Methods

### 2.1. Chemical Synthesis and Analysis

All reagents and anhydrous solvents were obtained from commercial sources and used without further purification except phenol distillation. Anhydrous solvents were dried over 4 Å molecular sieves and checked using a Karl Fisher titrator to determine if the water concentration was below 12 ppm before use. The progress of the chemical reactions was monitored by thin layer chromatography (TLC) on silica gel 60 F254 plates (Merc, Darmstadt, Germany). Spots on the TLC plate were visualized under UV light at 254 nm or by heating the plate after treatment with ninhydrin reagent made by dissolving 1.5 g of ninhydrin in 100 mL of *n*-butanol and adding 3.0 mL of acetic acid. Column chromatography was performed on Merck silica gel 60 (40–63 µm). Recycling preparative HPLC (prep-HPLC) was performed on a JAI LaboACE 5060 (Japan Analytic Industry, Tokyo, Japan). Depending on the type of compound to be purified, a tandem set of GPC JAIGEL-2HR+2.5 HR columns (∅20 mm × 600 mm) or a silica-based RP JAIGEL-ODS-AP-L SP-120-10 column (∅20 mm × 500 mm, 10 μm) was used for prep-HPLC. ^1^H-NMR and ^13^C-NMR spectra were recorded on a Varian NMR system 600 spectrometer (Agilent Technologies, Santa Clara, CA, USA) at 600 and 150 MHz, respectively. Peak multiplicity is expressed as follows: s = singlet, d = doublet, t = triplet, q = quartet, dd = doublet of doublets, ddd = doublet of doublets of doublets, m = multiplet. NMR chemical shifts are reported in ppm (δ), relative to residual nondeuterated solvents as internal standard and coupling constants (*J*) are given in Hz. Melting points (Mp) were determined using a Boethius microscope HMK type (Franz Küstner, Dresden, Germany). High-resolution electrospray ionization mass spectroscopy (HR-ESI-MS) analyses were performed on a Waters Xevo G2 QTOF apparatus (Waters-Micromass, Manchester, UK). Microwave-assisted reactions were carried out in a Biotage Initiator microwave reactor (Stockholm, Sweden) using 0.5–2.0 mL vials under the following conditions: 2 h, 90 °C, prestirring 30 s, high adsorption.

### 2.2. Ultraviolet Thermal Melting Studies

To determine the melting temperature (*T*_m_) of the obtained duplexes, UV melting studies were performed on a Lambda 35 UV/Vis Spectrometer (Perkin-Elmer, Norwalk, CT, USA) using 10 mm path length Hellma SUPRASIL quartz cuvettes (Müllheim, Germany), monitoring at 260 nm with a complementary DNA/DNA strands concentration of 2.5 μM and a volume of 1.0 mL. Samples were prepared as follows: The modified strands and their corresponding complementary strands were mixed 1:1 (*n/n*) in 2.0 mL Eppendorf tubes before medium salt buffer (2×, 11.7 mM sodium phosphate, pH 7.0, 200 mM NaCl, 0.20 mM EDTA, pH 7.0, 500 μL) was added, which was completed in 1.0 mL using Milli-Q water. Thus, all samples were dissolved in 1× buffer condition (5.8 mM sodium phosphate, pH 7.0, 100 mM NaCl, and 0.10 mM EDTA). The samples were denatured by heating to 90 °C in a water bath and then slowly cooled to rt before transferring them to cuvettes. The absorbance at 260 nm was recorded as a function of time with a linear temperature increase from 6 to 80 °C at a rate of 1.0 °C/min programmed by a Peltier temperature controller. Two separate melting curves were measured, and *T*_m_ values were calculated with the UV-WinLab software, taking the mean of the two melting curves with a deviation of no more than 0.5 °C.

### 2.3. Circular Dichroism Studies

Samples were prepared in the same way as for the *T*_m_ measurement. The background spectrum of the buffer was recorded and subtracted from the corresponding spectra. Measurements were performed on a JASCO J-815 spectrometer (Tokyo, Japan) at 20 °C using quartz optical cells with a path length of 5 mm and a total volume of 1.0 mL. All CD spectra were recorded from 200–400 nm with a scan rate of 100 nm/min, employing 5 scans.

### 2.4. Synthesis of 1,1′,1″-(1,4,7,10-Tetraazacyclododecane-1,4,7-triyl)tris(2,2,2-trifluoroethan-1-one); 1

TFAEt (18.0 mL, 150 mmol) was added dropwise to a stirred solution of cyclen (6.55 g, 38.0 mmol) and Et_3_N (5.27 mL, 38.0 mmol) in MeOH (40 mL) at rt for 30 min and left at rt overnight. All volatiles were evaporated in vacuo and the residual oil was suspended in AcOEt (10 mL), evaporated onto 10 times its weight of silica gel and purified by silica gel column (∅50 mm × 200 mm), eluting with 100% AcOEt. The fractions at R_f_ 0.35 (TLC, 100% AcOEt), which became slightly stained in ninhydrin reagent, were evaporated to give **1** as a white foam (15.7 g, 85%). Mp = 79–80 °C. ^1^H-NMR (600 MHz, DMSO-d_6_): δ 3.91–3.78 (m, 4H), 3.66–3.42 (m, 8H), 2.82–2.67 (m, 4H), 2.33 (q, *J* = 8.3 Hz, 1H). ^13^C-NMR (150 MHz, DMSO-d_6_): δ 156.78–156.24 (m), 117.53 (q, *J* = 286.4 Hz), 55.28–43.00 (m). HR-ESI-MS: *m*/*z* calcd. for C_14_H_18_F_9_N_4_O_3_ 461.1235 [M+H]^+^; found 461.1134. The proton-decoupled ^13^C-NMR spectra of TFA-protected cyclen and its derivatives are complicated by the C–F coupling and the presence of conformers at rt.

### 2.5. Synthesis of 1,1′,1″-(10-(5-Bromopentanoyl)-1,4,7,10-tetraazacyclododecane-1,4,7-triyl)tris(2,2,2-trifluoroethan-1-one); 2

An amount of 5-Bromovaleryl chloride (562 μL, 4.23 mmol) was added to a stirred solution of **1** (1.77 g, 3.84 mmol) in DCM (20 mL) with K_2_CO_3_ (585 mg, 4.23 mmol) and stirred for 40 min in an ice-water bath. The progress of the reaction was monitored by TLC (5% MeOH-CHCl_3_, *v/v*) for the appearance of a new spot at R_f_ 0.42, which turned pale brown after ninhydrin treatment, and disappearance of the substrate. After the substrate spot was completely consumed, the reaction mixture was washed with water (20 mL) and the organic layer was dried over Na_2_SO_4_. The filtrate was evaporated, and the oil residue was purified on a silica gel column (∅15 mm × 400 mm) eluting with 50% AcOEt-hexane (*v/v*). The fractions at R_f_ 0.22 (TLC, 50% AcOEt-hexane, *v/v*) were combined and evaporated to give **2** as a viscous oil (1.94 g, 81%). ^1^H-NMR (600 MHz, DMSO-d_6_): δ 3.84–3.67 (m, 16H), 3.55 (t, *J* = 6.6 Hz, 2H), 2.38–2.31 (m, 2H), 1.85–1.81 (m, 2H), 1.65–1.63 (m, 2H). ^13^C-NMR (150 MHz, DMSO-d_6_): δ 172.92, 156.59–156.01 (m), 116.04 (q, *J* = 286.5 Hz), 48.02–45.11 (m), 40.04, 34.74, 31.72, 23.23, 23.16.

### 2.6. Synthesis of 1,1′,1″-(10-(5-Azidopentanoyl)-1,4,7,10-tetraazacyclododecane-1,4,7-triyl)tris(2,2,2-trifluoroethan-1-one); 3

NaN_3_ (130 mg, 2.00 mmol) was added to a solution of **2** (623 mg, 1.00 mmol) in DMF (10 mL) and stirred for 24 h at rt. TLC analysis (50% AcOEt-hexane, *v/v*) showed a new spot of **3** at R_f_ 0.45, near the substrate at R_f_ 0.40, which stains darker on heating with ninhydrin than the substrate spot. The mixture was partitioned between AcOEt (20 mL) and water (80 mL), the organic layer was dried over Na_2_SO_4_ and evaporated to give a viscous oil, which was purified by prep-HPLC (JAIGEL-ODS-AP-L, 100% MeOH, flow rate 7.0 mL/min). The fraction at a *t*_R_ 26.8 min was collected and evaporated to give **3** (115 mg, 94%). ^1^H-NMR (600 MHz, CD_3_OD): δ 3.89–3.73 (m, 16H), 3.34 (t, *J* = 6.6 Hz, 2H), 2.46–2.42 (m, 2H), 1.71–1.69 (m, 2H), 1.65–1.62 (m, 2H). ^13^C-NMR (150 MHz, CD_3_OD): δ 174.88, 157.84–157.05 (m), 116.27 (q, *J* = 285.8), 50.79, 47.07–46.28 (m), 32.12, 28.01, 22.05.

### 2.7. Synthesis of 2-Hydroxy-9H-thioxanthen-9-one; 4

Freshly distilled phenol (9.00 g, 97.3 mmol) was added portionwise to a suspension of thiosalicylic acid (5.00 g, 32.4 mmol) at concd. H_2_SO_4_ (96%, 60 mL) and the mixture was heated at 90 °C for 18 h. After cooling to rt, the mixture was gently poured into 500 mL of water with crushed ice to give a yellow precipitate, which was filtrated off and dried to give a yellow solid. The crude solid was dissolved in CHCl_3_ (50 mL) and evaporated onto 10 times its weight of silica gel, applied to a silica gel column (∅50 mm × 200 mm), and eluted using 5% MeOH-CHCl_3_ (*v/v*). The fractions visible on TLC as yellow spots at R_f_ 0.32 was evaporated together to give **4** as a yellow solid (3.41 g, 46%). Mp = 245–246 °C. ^1^H-NMR (600 MHz, CD_3_OD): δ 10.22 (s, 1H), 8.52 (ddd, *J* = 7.8, 1.2, 0.6, 1H), 7.93 (d, *J* = 2.4, 1H), 7.65-7.70 (m, 2H), 7.57 (d, *J* = 9.0, 1H), 7.50 (ddd, *J* = 8.4, 6.0, 1.8, 1H), 7.25 (dd, *J* = 9.0, 3.0, 1H). ^13^C-NMR (150 MHz, CD_3_OD): δ 179.30, 156.53, 137.98, 137.45, 132.08, 129.86, 129.00, 128.12, 127.29, 125.90, 125.75, 122.32, 113.12. HR-ESI-MS: *m*/*z* calcd. for C_13_H_7_O_2_S 227.0172 [M-H]^−^; found 227.0167.

### 2.8. Synthesis of 2-(4-Bromobutoxy)-9H-thioxanthen-9-one; 5

An amount of 1,4-dibromobutane (1.40 mL, 11.9 mmol) was added in one portion to a mixture of K_2_CO_3_ (900 mg, 56.6 mmol) and **4** (680 mg, 2.98 mmol) in DMF (15 mL) and stirred at 100 °C for 48 h. The mixture was cooled to rt, diluted with AcOEt to 40 mL, and washed with water (100 mL). The organic layer was dried over Na_2_SO_4_, filtered off, and evaporated to give a yellow oil, which crystallized over time. The crude solid was purified by silica gel chromatography (∅15 mm × 450 mm) eluting with 100% CHCl_3_. The fractions at R_f_ 0.75 (TLC, 100% CHCl_3_) were evaporated to give **5** as a light-yellow solid (922 mg, 85%). Mp = 122–123 °C. ^1^H-NMR (600 MHz, CDCl_3_): δ 8.61 (ddd, *J* = 7.8, 1.2, 0.6 Hz, 1H), 8.04 (d, *J* = 2.4 Hz, 1H), 7.60 (ddd, *J* = 7.2, 6.6, 1.2 Hz, 1H), 7.57 (ddd, *J* = 8.4, 1.8, 0.6 Hz, 1H), 7.48 (d, *J* = 9.0 Hz, 1H), 7.47 (ddd, *J* = 7.2, 6.6, 1.2 Hz, 1H), 7.24 (dd, *J* = 8.4, 2.4 Hz, 1H), 4.13 (t, *J* = 6.0 Hz, 2H), 3.50 (t, *J* = 6.6 Hz, 2H), 2.19–2.07 (m, 2H), 2.02–1.97 (m, 2H). ^13^C-NMR (150 MHz, CDCl_3_): δ 179.59, 157.62, 137.47, 131.99, 130.20, 129.85, 129.14, 128.57, 127.29, 126.05, 125.95, 122.88, 111.08, 67.33, 33.29, 29.44, 27.78.

### 2.9. Synthesis of 2-(4-Azidobutoxy)-9H-thioxanthen-9-one; 6

The reaction of NaN_3_ (130 mg, 2.00 mmol) with solution of **5** (363 mg, 1.00 mmol) in DMF (15 mL) was performed similar to that of **3**. The crude product was purified on a silica gel column (∅15 mm × 450 mm) and the eluates at R_f_ 0.95 (100% CHCl_3_), turning grey on heating with ninhydrin, were evaporated and purified by prep-HPLC (JAIGEL-2HR+2.5HR, 100% DCM, flow rate 7.0 mL/min). The fraction at a *t*_R_ 36.3 min was evaporated to give **6** (310 mg, 95%) as a yellow oil, which crystallized over time. Mp = 77–78 °C. ^1^H-NMR (600 MHz, DMSO-d_6_): δ 8.46 (ddd, *J* = 8.4, 1.8, 0.6 Hz, 1H), 7.89 (d, *J* = 3.0 Hz, 1H), 7.80 (ddd, *J* = 8.4, 1.8, 0.6 Hz, 1H), 7.75 (ddd, *J* = 7.2, 6.6, 1.2 Hz, 1H), 7.73 (d, *J* = 9.0 Hz, 1H), 7.57 (ddd, *J* = 7.2, 6.6, 1.2 Hz, 1H), 7.39 (dd, *J* = 8.4, 3.0 Hz, 1H), 4.12 (t, *J* = 6.0 Hz, 2H), 3.44 (t, *J* = 6.6 Hz, 2H), 1.84–1.81 (m, 2H), 1.76–1.71 (m, 2H). ^13^C-NMR (150 MHz, DMSO-d_6_): δ 178.80, 157.80, 137.19, 133.07, 129.86, 129.48, 128.58, 128.39, 128.16, 126.90, 126.87, 123.02, 111.46, 67.84, 50.85, 26.28, 25.51.

### 2.10. Synthesis of 2-(4-Bromobutyl)-1H-benzo[de]isoquinoline-1,3(2H)-dione; 7

To a stirred suspension of 1,8-naphthalimide (1.12 g, 5.68 mmol) in dry DMF (20 mL), NaH (80% dispersion in mineral oil; 341 mg, 11.36 mmol) was added portionwise. The suspension was allowed to stir at rt for 1 h. Then, 1,4-dibromobutane (2.37 mL, 22.72 mmol) was added to the reaction mixture in one portion and stirred overnight at rt. The reaction mixture was then poured into a 5% HCl solution and the resulting white precipitate was filtered off and air dried. The crude solid was purified by silica gel chromatography (∅15 mm × 450 mm) eluting with 100% CHCl_3_. Fractions with R_f_ 0.90 (TLC, 100% CHCl_3_) were evaporated to afford **7** as a white solid (1.40 g, 74%). Mp = 115–116 °C. ^1^H-NMR (400 MHz, CDCl_3_): δ 8.58 (dd, *J* = 7.2, 1.2 Hz, 2H), 8.21 (dd, *J* = 8.4, 1.2 Hz, 2H), 7.75 (dd, *J* = 8.2, 7.4 Hz, 2H), 4.22 (t, *J* = 7.0 Hz, 2H), 3.48 (t, *J* = 6.6 Hz, 2H), 1.96-2.03 (m, 2H), 1.86–1.95 (m, 2H). ^13^C-NMR (100 MHz, CDCl_3_): δ 164.11, 133.92, 131.53, 131.24, 128.11, 126.96, 122.57, 39.30, 33.11, 30.22, 26.94.

### 2.11. Synthesis of 2-(4-Azidobutyl)-1H-benzo[de]isoquinoline-1,3(2H)-dione; 8

The reaction of NaN_3_ (474 mg, 7.28 mmol) with solution of **7** (1.21 g, 3.64 mmol) in DMF (20 mL) was performed similar to that of **3**. The crude product was purified on a silica gel column (∅15 mm × 450 mm) and the eluates at *R*_f_ 0.95 (100% CHCl_3_), turning grey on heating with ninhydrin, were evaporated and purified by prep-HPLC (JAIGEL-2HR + 2.5HR, 100% DCM, flow rate 7.0 mL/min). The fraction at a *t*_R_ 32.1 min was evaporated to give **8** (980 mg, 91%) as a white solid. Mp = 73–74 °C. ^1^H-NMR (600 MHz, CDCl_3_): δ 8.58 (dd, *J* = 7.2, 1.1 Hz, 2H), 8.20 (dd, *J* = 8.3, 1.0 Hz, 2H), 7.76–7.73 (m, 2H), 4.21 (t, *J* = 7.4 Hz, 2H), 3.35 (t, *J* = 6.9 Hz, 2H), 1.86–1.81 (m, 2H), 1.74–1.69 (m, 2H). ^13^C-NMR (150 MHz, CDCl_3_): δ 164.14, 133.93, 131.56, 131.23, 128.12, 126.90, 122.56, 51.16, 39.61, 26.49, 25.37.

### 2.12. Oligonucleotides Purification and Analysis

RP-HPLC purification of crude oligonucleotides was performed by Waters 600 HPLC System with a Waters XBridge BEH C18-column (∅19 mm × 100 mm, 5 μm). Elution was performed by isocratic hold of A-buffer for 5.0 min, followed by a linear gradient to 70% of B-buffer for 16.5 min at a flow rate of 5.0 mL/min (A-buffer: 0.05 M TEAA buffer, pH 7.4; B-buffer: 25% A-buffer, 75% MeCN). IE-HPLC purification of oligonucleotides was caried on a DIONEX Ultimate 3000 system with a DNAPac PA100 Semi-Preparative column (∅9 mm × 250 mm, 13 μm) at 60 °C (Thermo Fisher Scientific, Darmstadt, Germany). Elution was performed with an isocratic hold of 10% C-buffer in Milli-Q water, starting with hold on 2% D-buffer in Milli-Q water for 2.0 min, followed by a linear gradient to 25% of D-buffer in Milli-Q water for 20.0 min at a flow rate of 2.0 mL/min (C-buffer: 0.25 M Tris-Cl, pH 8.0; D-buffer: 1.0 M NaClO_4_). After purification, the appropriate fractions were combined and concentrated by purging with N_2_ at 55 °C, and the obtained samples were dissolved in Milli-Q water (100 µL), then desalted with an addition of NaClO_4_ solution (5.0 M, 15 µL), suspended in cold ethanol (1.5 mL) and stored at −20 °C for 1–2 h. After centrifugation (13,200 rpm, 5 min, 4 °C), the supernatant was filtered off and the pellet was washed with cold ethanol (2 × 1.0 mL), dried under N_2_ flow at 55 °C, and dissolved in Milli-Q water (1.0 mL). Analytical RP-HPLC was performed on a Merck-Hitachi 7000 system (Hitachi Instruments, Tokyo, Japan) equipped with a Waters XBridge OBD C18-column (∅10 mm × 50 mm, 2.5 µm) at 60 °C. Elution was started with an isocratic hold of A-buffer for 2 min followed by a linear gradient to 85% of B-buffer for 30 min, keeping the flow rate at 1.3 mL/min. The structure and composition of oligonucleotides was verified by the MALDI-TOF MS method performed on an Ultraflex Extreme mass spectrometer (Bruker Daltonics, Bremen, Germany). Finally, the purified oligonucleotides were quantified by measuring OD as the absorbance at 260 nm of the sample in 1.0 mL of water in a 10 mm path length cuvette. The excitation coefficients for DNAs at 260 nm were estimated to be 1 × 10^4^ M cm^−1^ residue^−1^.

### 2.13. Synthesis and Purification of ON1–ON5

Target **ON1**–**ON5** were synthesized at the 1.0 μmol scale on polystyrene beads (Amersham Biosciences, Piscataway, NJ, USA) using an automated synthesizer Expedite 8909 (PerSeptive Biosystems, Framingham, MA, USA) according to the manufacturer’s standard protocol, except for the introduction of 2′-*O*-propargyl-uridine (U^P^) into the **ON3**–**ON5** sequence by the so-called “hand-coupling procedure”, previously used by Wengel’s group [17]. The stepwise coupling efficiencies were >95% for standard conditions and ∼85% for hand-coupling. Cleavage from the beads and nucleobase deprotection were performed by incubation with concd. aq. NH_3_ in a screw cap vial at 55 °C overnight. The supernatant was filtered and evaporated to remove NH_3_ by heating the filtrate to 55 °C and purging with N_2_ for 4 h. The crude samples were purified DMT-on by RP-HPLC and the 5′-DMT group was cleaved with 2% aq. trifluoroacetic acid. The deprotected oligonucleotides were eluted with a 30% MeCN soln. in water (*v/v*) and purified by IE-HPLC, then the composition of the collected fractions was assessed by MALDI-TOF MS. Unmodified and 2′-*O*-propargylated oligonucleotides were isolated in overall yields of 80–88% and were >98% pure by IE-HPLC analysis.

### 2.14. Synthesis and Purification of ON9-ON12, ON13 and ON14

To a 2.0 mL Ar purged microwave vial containing **ON3** (203 nmol in 800 μL of dH_2_O) in a mixture of TEAA buffer (250 μL, 1.0 M, pH 7.4) and DMSO (400 μL), azide-functionalized **3** (51.0 μL, 10.0 mM DMSO soln.), freshly prepared CuSO_4_–TBTA equimolar complex (80.0 μL, 10.0 mM DMSO-dH_2_O mixture, 3:7, *v/v*) and sodium ascorbate (201 μL, 25.0 mM dH_2_O soln.) were subsequently added. The resulting mixture was vortexed and centrifuged after adding each of the reagents. The vial was equipped with a magnetic stirrer, purged with Ar, sealed with a Teflon-lined septum cap, and microwaved. After completion of the reaction, the volume was made up to 2.0 mL with dH_2_O and divided into two equal parts. Each sample was desalted through a NAP-10 column (GE Healthcare, Little Chalfont, UK) following manufacturer’s protocol. The resulting solution contains a mixture of two major products, tris-*N*-TFA-protected **ON6** and partially deprotected bis-*N*-TFA-protected **ON6′**. During RP-HPLC purification, the fractions ranging from *t*_R_ 12.2 to 17.2 min were collected, evaporated together under a stream of N_2_, and used as a mixture in the next step. The resulting sample was incubated with 1.0 mL of satd. aq. NH_3_ at 55 °C overnight and then evaporated by gentle blowing with N_2_ at 30 °C for 4 h. The crude sample after deprotection was purified by IE-HPLC to give **ON13** in 41% overall yield and 98% purity.

The synthesis of the intercalator-labeled **ON9**–**ON12** and cyclen-labeled **ON14** was performed under the same conditions as for **ON13**. After desalting through an NAP-10 column, **ON9**–**ON12** samples were evaporated under a stream of N_2_ and purified by RP-HPLC. These samples were obtained in high yield and purity (Table 1) and did not require further purification by IE-HPLC. After coupling **3** with **ON4**, a mixture of intermediates **ON7”** was obtained which was purified by RP-HPLC by collecting the fractions at *t*_R_ from 12.2 to 17.2 min. After their joint deprotection and purification of the resulting sample by IE-HPLC, the **ON14** conjugate was obtained in an overall yield of 40% and 98% purity.

### 2.15. Synthesis and Purification of ON15

For the synthesis of the double-functionalized **ON15**, the same procedure was used as for **ON13**, except that the following reaction system was used: **ON5** (83 nmol in 480 μL of dH_2_O) in a mixture of TEAA buffer (150 μL, 1.0 M, pH 7.4) and DMSO (240 μL), azide-functionalized **3** (42.0 μL, 10.0 mM DMSO soln.), freshly prepared CuSO_4_–TBTA equimolar complex (33.0 μL, 10.0 mM DMSO–dH_2_O mixture, 3:7, *v/v*) and sodium ascorbate (164 μL, 25.0 mM dH_2_O soln.). The fractions ranging from *t*_R_ 12.7 to 17.5 min were collected by RP-HPLC and evaporated together under a stream of N_2_. After complete deprotection, a single peak was observed at *m*/*z* 3428.227 in the MALDI-TOF MS spectra assigned to **ON15** (calcd. as *m*/*z* 3428.957) and a single peak in the RP-chromatogram at *t*_R_ 8.39 min. This fraction was collected and purified by IE-HPLC to give **ON15** in 38% overall yield and 95% purity.

## 3. Results

### 3.1. Chemical Synthesis of Labels

**Scheme 1:**
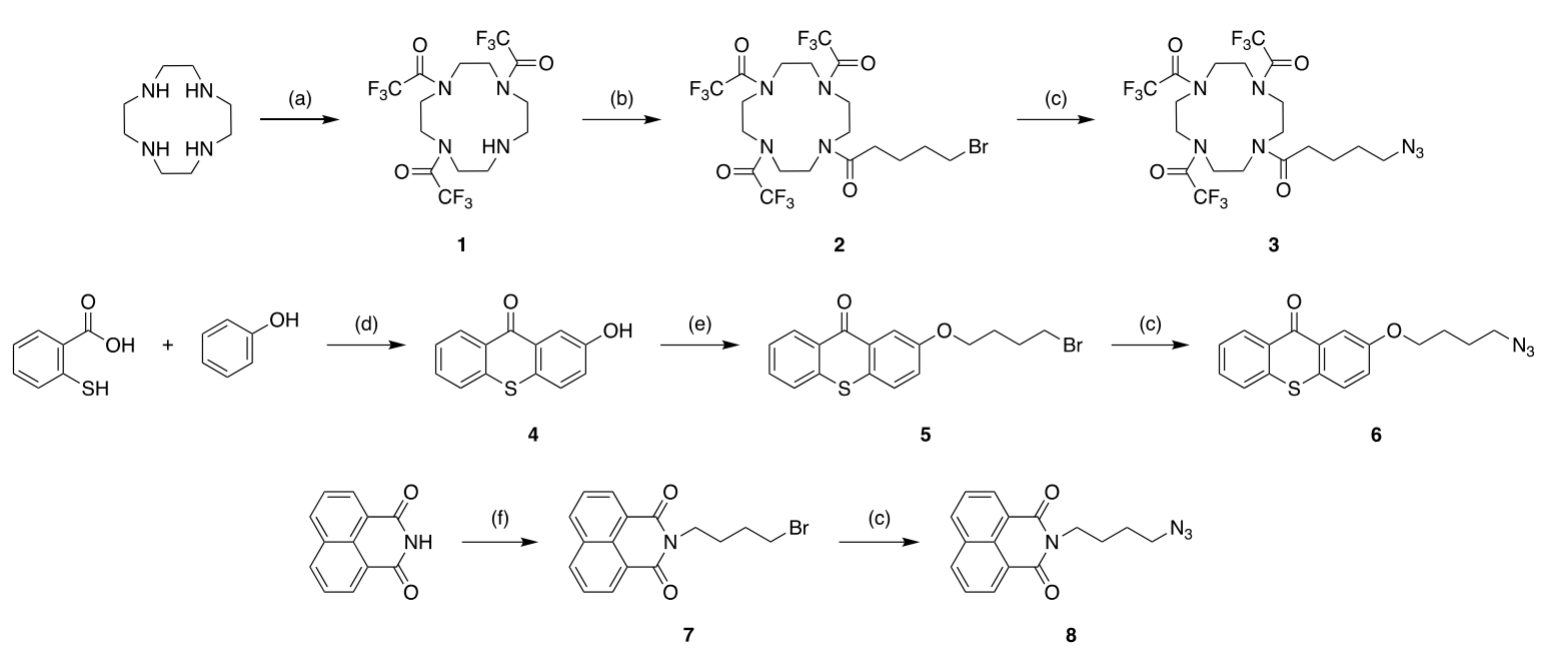
Synthesis of azide-functionalized labels. Reagents and conditions: (a) TFAEt, Et_3_N, MeOH, rt, overnight; (b) 5-bromovaleryl chloride, K_2_CO_3_, dry CH_2_Cl_2_, 0 °C, 40 min; (c) NaN_3_, DMSO, rt, 24 h; (d) concd. H_2_SO_4_, 90 °C, 18 h; (e) 1,4-dibromobutane, K_2_CO_3_, DMF, 90 °C, 48 h; (f) NaH, dry DMF, rt, 1 h; then 1,4-dibromobutane, rt, overnight.

The structures of cyclen and selected intercalating dyes do not provide suitable functional groups for direct attachment to oligonucleotides, so we first synthesized their derivatives having an azide-terminated linker. The synthesis is shown in Scheme 1 and performed according to well-known methods with some modifications. First, three of the four cyclen amino groups were selectively *N*-protected as trifluoroacetamides (*N*-TFA) using ethyl trifluoroacetate (TFAEt) and purified by column chromatography in accordance with the method described previously [20]. The trifluoroacetamide protecting groups were chosen because of their easy and efficient removal in the last step of conjugate synthesis. During further steps, amine **1** was reacted with commercially available 5-bromovaleryl chloride in dry CH_2_Cl_2_, followed by treatment with NaN_3_ in DMSO to form azide-terminated **3** with a 65% overall yield. Then, 2-Hydroxy-9*H*-thioxanthen-9-one **4** was synthesized by the reaction of phenol with thiosalicylic acid, which proceeds through successive EAS reactions and culminates in intramolecular Friedel–Crafts cyclization to form a tricyclic thioxanthone core [21]. Reaction of **4** and 1,8-napthalimide with 1,4-dibromobutane led to **5** and **7**, respectively, in good yields. Further substitution of the terminal bromine for the azide group led to the formation of target compounds **6** and **8** with overall yield of 37 and 76%, respectively. The introduced linkers are expected to move the labels far enough and provide them sufficient flexibility to be close to the duplex backbone. The final products were purified by preparative HPLC before their conjugation with oligonucleotides.

### 3.2. Synthesis and Modification of Oligonucleotides

**Scheme 2:**
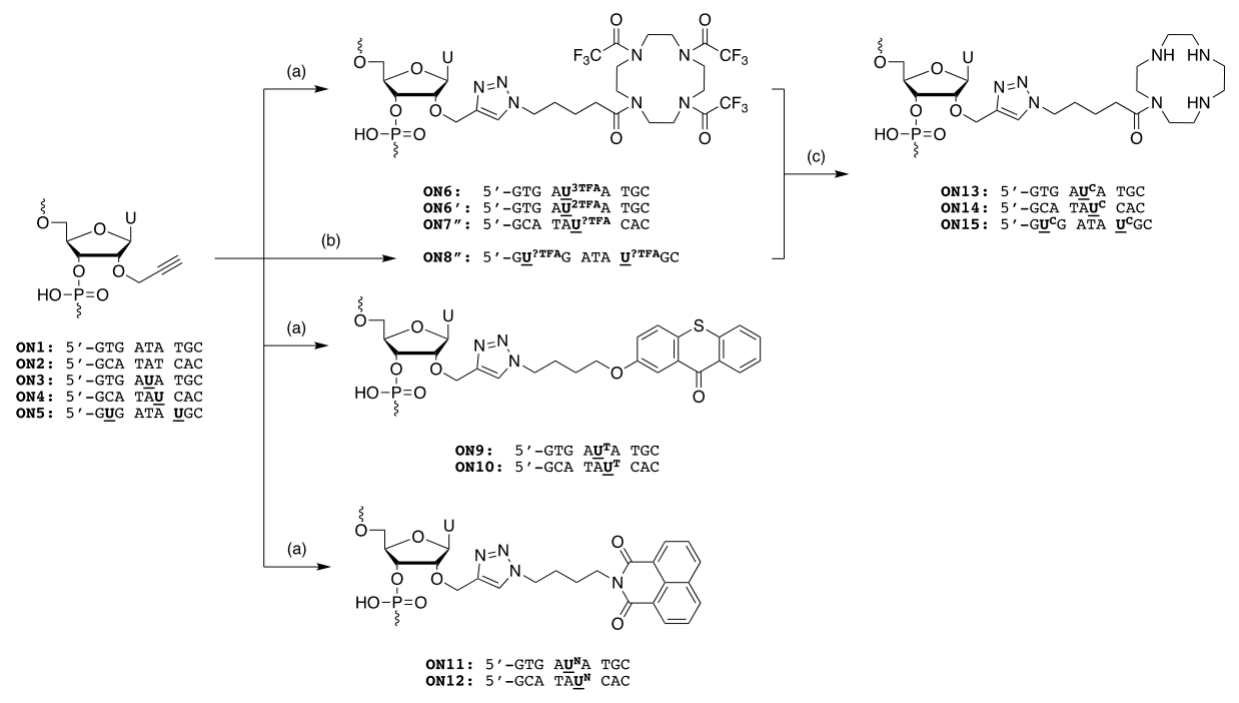
A post-synthetic approach for the synthesis of 2′-labeled oligonucleotides. Reagents and conditions: (**a**) CuSO_4_–TBTA, sodium ascorbate (*n/n* 1:1:2.5), TEAA buffer/DMSO, Ar, microwave, 90 °C, 2 h; (**b**) CuSO_4_–THPTA complex, sodium ascorbate (*n/n* 1:1:2.5), TEAA buffer/DMSO, Ar, microwave, 90 °C, 2 h; (**c**) concd. aq. NH_3_ (35%), 55 °C, overnight. 3TFA: tris-*N*-TFA-protected cyclen; 2TFA: bis-*N*-TFA-protected cyclen, ?TFA: unknown degree of cyclen protection. **ON6‘**: RP-HPLC separated fraction of the conjugate with bis-*N*-TFA-protected cyclen moiety. **ON7”**, **ON8”**: Unseparated mixture of conjugates with differentially *N*-TFA-protected cyclen moieties.

Scheme 2 shows a labeling method by post-synthetic coupling of azide-functionalized labels to 2′-*O*-propargylated oligonucleotides using Cu(I)-catalyzed azide–alkyne cycloaddition (CuAAC). Target oligonucleotides were prepared at the 1.0 µmol scale using an automated synthesizer and standard solid phase phosphoramidite chemistry. The 2′-*O*-propargyl-U (U^P^) units were incorporated into the growing chains of **ON3**–**ON5** in a sequence-specific manner using the so-called “hand-coupling protocol”, which involves manual injection of U^P^ phosphoramidite and increasing the coupling time to 25 min [22]. Covalent functionalization of mono-alkyne-modified **ON3** and **ON4** was carried out by microwave-assisted CuAAC using 0.4-fold molar ratio of CuSO_4_–TBTA complex with sodium ascorbate (*n/n*, 1:1:2.5) and a 4-fold ratio of azide-functionalized label to oligonucleotide [23]. Using the same reaction conditions to functionalize the di-alkyne analogue, **ON5**, did not give the expected double-clicked adduct, **ON8”**, even after doubling the concentration of the catalyst system and azide label and increasing the MW reaction time to 6 h. We then sought to determine whether Cu(I)–THPTA or Cu(I)–BPS complexes could force the reaction to the double-clicked adduct better than Cu(I)–TBTA. We found that using THPTA instead of TBTA and BPS as a ligand, at the same molar ratio of the catalytic system, could promote the dual coupling of **ON5**, but showed no improvement in the single coupling of **ON3** or **ON4**.

**Figure 1:**
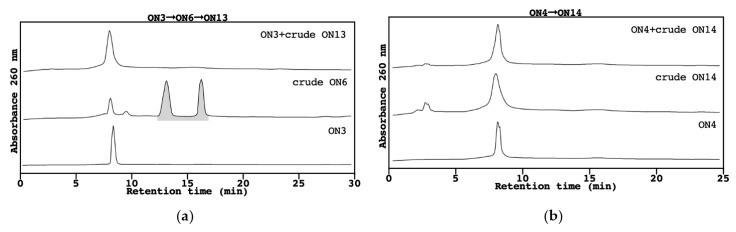

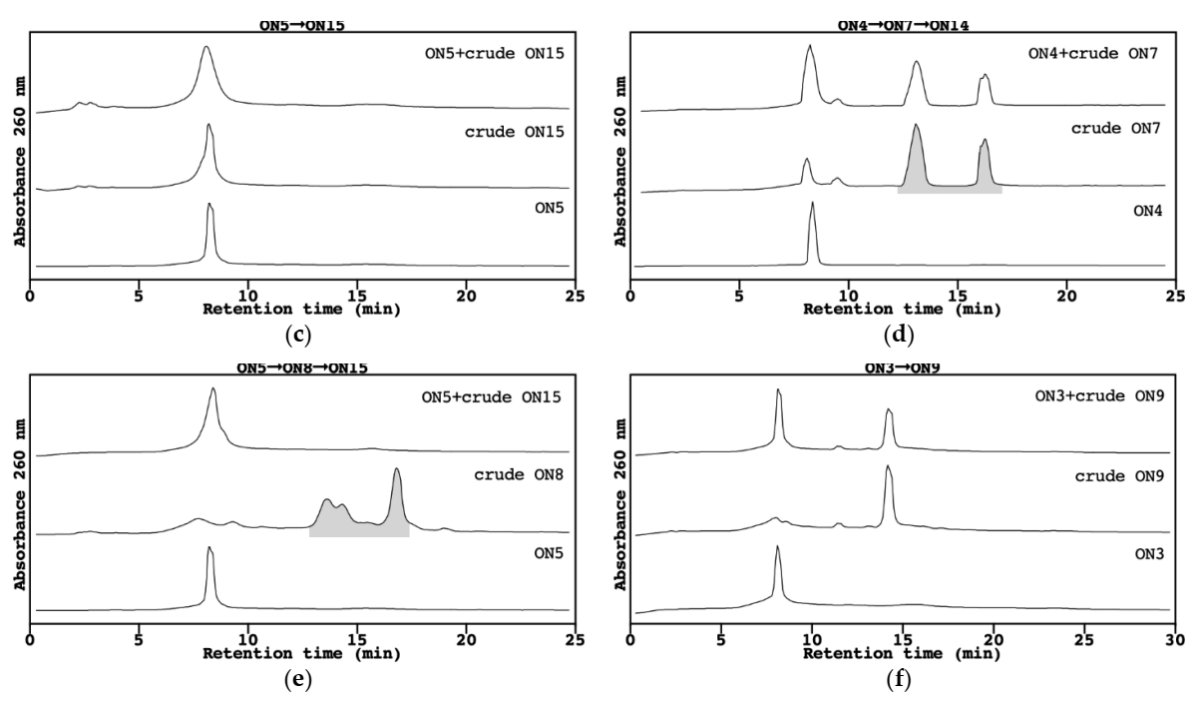
The overlay of analytical RP-HPLC chromatograms from the subsequent stages of the synthesis of modified oligonucleotides: (**a**) **ON13** by separation of **ON6”**, (**b**) **ON14** without separation of **ON7”**, (**c**) **ON15** without separation of **ON8”**, (**d**) **ON7”**, (**e**) **ON15** by separation of **ON8”**, (**f**) **ON9**. RP-HPLC conditions: XBridge BEH C18-column (∅19 mm × 100 mm, 5 μm); mobile phase: A: 0.05 M TEAA buffer, pH 7.4; B: 25% A, 75% MeCN; isocratic hold of A for 5.0 min, followed by a linear gradient to 70% B for 16.5 min; flow rate 5.0 mL/min; rt.

RP-HPLC analysis of the crude sample obtained after conjugation of **3** with **ON3** followed by desalting on a NAP-column show the presence of two new fractions (shaded in gray in Figure 1a), well separated from each other and from the starting **ON3**. We collected both fractions separately and found that the one at *t*_R_ 13.1 min gives a signal at *m*/*z* 3283.192, corresponding to **ON6′** (calcd. as *m*/*z* 3283.295) with a partially deprotected cyclen moiety, while the fraction at *t*_R_ 16.3 min gives a signal at *m*/*z* 3379.920, corresponding to **ON6** (calcd. as *m*/*z* 3379.303) with a fully protected cyclen moiety. Regardless of whether these fractions were collected and deprotected separately or together, in the RP-chromatogram, we observed the presence of only one fraction at *t*_R_ 8.4 min with a signal at *m*/*z* 3091.684, coming from **ON13** (calcd. as *m*/*z* 3091.278) having a fully deprotected cyclen moiety. After final IE-HPLC purification, **ON13** was obtained in a total yield of 41% and a purity of 98% by IE-analysis (Supplementary Materials Figure S1).

The overlay of RP-chromatograms in Figure 1a–e shows that omitting the isolation of the *N*-TFA-protected cyclen–oligonucleotide conjugates from the crude **ON6**–**ON8** samples and proceeding directly to the deprotection step prevented further separation of the fully deprotected conjugates from the starting alkynylated oligonucleotides by RP-HPLC. In all cases, the retention time of the fully deprotected conjugates is almost equal to that of the starting alkynylated oligonucleotides and only a single peak is visible after the coinjection of both samples. IE-HPLC analysis of **ON14** obtained by this procedure showed at least 17% content of the reaming fractions (Figure S2). In turn, when **ON14** was prepared by the same procedure as for **ON13**, i.e., by collecting fractions of differently protected **ON7″** conjugates (shaded in gray in Figure 1d) and deprotecting them together, we could easily obtain the final product in 41% overall yield and significant higher 98% purity (Figure S3). We then applied this procedure to obtain **ON15** by bifunctionalization of **ON5**, which, unlike monofunctionalization, resulted in a mixture of several overlapping fractions of differentially protected **ON8″** conjugates, seen in the RP-chromatogram at *t*_R_ of 12.5 to 18.2 min (shaded in gray in Figure 1e). When these fractions were collected and deprotected together (Figure S4), a single fraction was observed by RP-analysis at *t*_R_ 8.4 min with a signal at *m/z* 3428.227 corresponding to **ON15** (calcd. as *m*/*z* 3428.957).

Coupling of the thioxanthone derivative **6** to **ON3** gave only one RP-fraction at *t*_R_ 14.4 min with a signal at *m*/*z* 3118.754 corresponding to **ON9** (calcd. as *m*/*z* 3119.595); this was accompanied by the disappearance of the initial **ON3** peak at *t*_R_ 8.4 min (Figure 1f). Purification by RP-HPLC gave **ON9** with a purity of 98% and an overall yield of 56%; according to IE-analysis, the sample was sufficiently pure to be used in further duplex stabilization studies without the need for additional IE-HPLC purification (Figure S5). A similar situation occurs for the conjugation of the 1,8-naphthalimide derivative **9** with **ON3** and for other intercalating dyes; the products of these reactions are well separated from the starting materials and, after RP-HPLC purification, can be used directly for further studies (see Supplementary Materials, Figures S6–S8 show the results of IE-analysis for the remaining conjugates).

### 3.3. Circular Dichroism Studies

**Figure 2:**
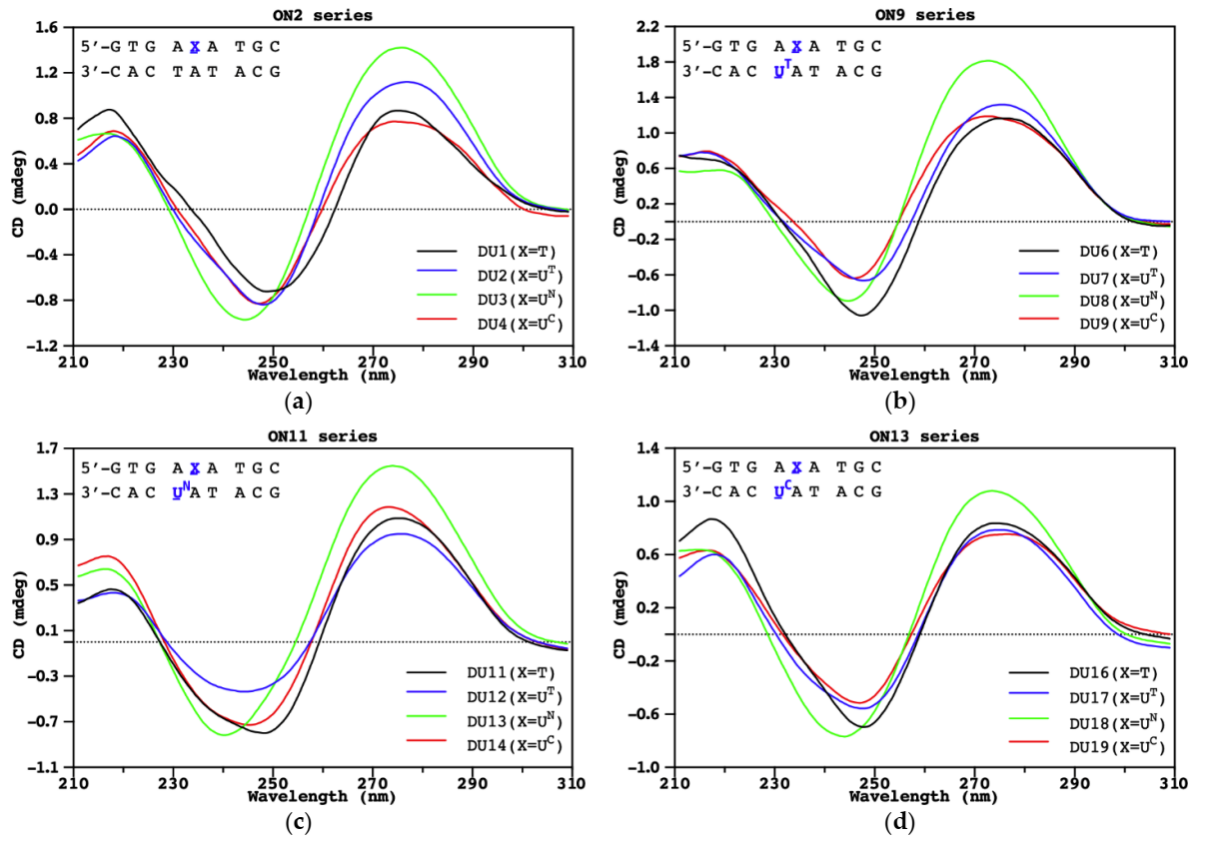
Circular dichroism spectra at rt of (**a**) **DU1**–**DU4**, (**b**) **DU6**–**DU9**, (**c**) **DU11**–**DU14,** and (**d**) **DU16**–**DU19**. The experiments were performed in a medium salt buffer 5.8 mM NaH_2_PO_4_/Na_2_HPO_4_ buffer (pH 7.0), containing 100 mM NaCl and 0.10 mM EDTA. The concentration of oligonucleotide: Watson–Crick complementary strand = 2.5:2.5 μM.

Figure 2 shows circular dichroism (CD) spectra recorded to find possible changes in the secondary structure of the labeled duplexes. For all of them, the CD spectra showed intense negative and positive amplitudes at ~250 nm and ~280 nm, respectively, with no major differences relative to unmodified **DU1** DNA duplex (black line in Figure 2a). The intensity of the bands also did not change significantly relative to unmodified **DU1**, suggesting than all modifications introduced do not induce any changes in the overall B-type duplex structure.

### 3.4. Ultraviolet Thermal Melting Studies

**Figure 3:**
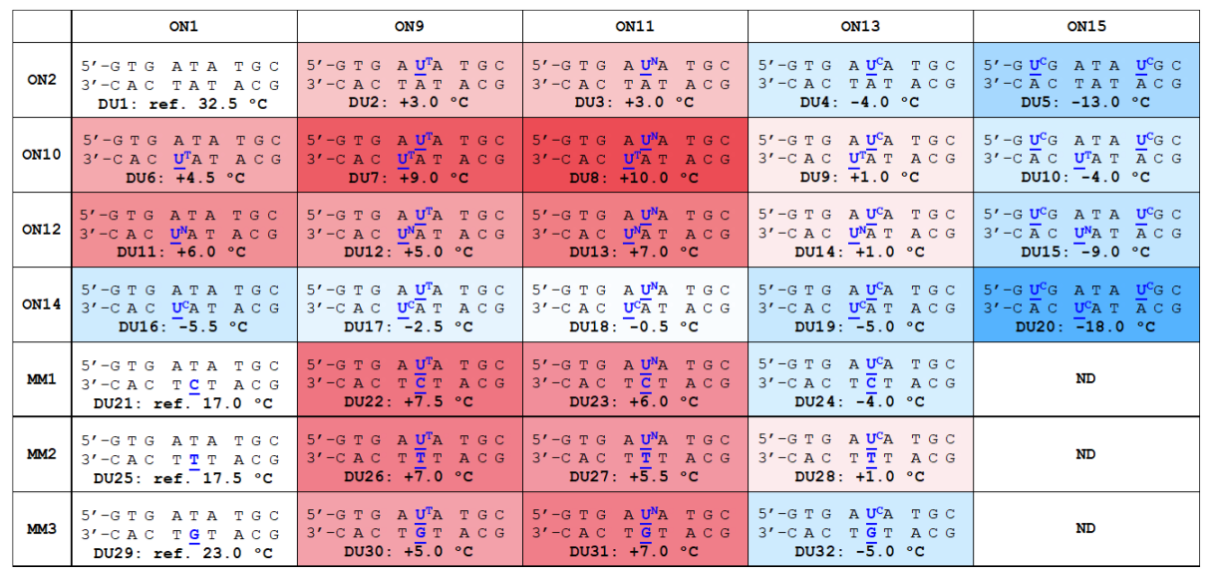
Heat map of the relative changes in melting temperatures (Δ*T*_m_) of the modified duplexes compared to their respective controls. Δ*T*_m_ values are the average of three measurements. **DU1** is reference for **DU2**–**DU20**, **DU21** is reference for **DU22**–**DU24**, **DU25** is reference for **DU26**–**DU28**, **DU29** is reference for **DU30**–**DU32**. Red indicates an increase in the thermal stability of the duplex, blue indicates a decrease. ND: not detected. Conditions: 2.5 μM of each strand in a medium salt buffer 5.8 mM NaH_2_PO_4_/Na_2_HPO_4_ buffer (pH 7.0), containing 100 mM NaCl and 0.10 mM EDTA. The *T*_m_ values reflect the average of two measurements. Δ*T*_m_ values for mismatches were calculated as the difference in *T*_m_ values between unmodified mismatched and modified mismatched duplexes.

Figure 3 summarizes the duplex sequences along with the relative changes in melting temperatures (Δ*T*_m_) compared to the corresponding references. The unmodified duplex **D1** has a reference *T*_m_ of 32.5 °C, which is consistent with literature data [24]. In general, oligonucleotides with attached intercalating dyes have a positive effect on the thermal stability of all duplexes obtained, especially duplexes containing mismatches on one of the strands. The magnitude of this effect depends on the position and type of intercalating dye attached; in the case of duplexes containing only one modified strand, the highest increase in melting temperature was observed for **DU6** and **DU11**, in which intercalator-labeled U^T^ and U^N^ were adjacent to the GC base pair. For most duplexes containing two intercalator-labeled strands, an additive stabilizing effect was observed, although its magnitude depended on the combination and position of the labeled nucleotides. The largest stabilizing effect was observed for **DU8**, for which Δ*T*_m_ is +10 °C. Interestingly, in the case of **DU12** with an interchanged dye arrangement, compared to **DU8**, despite the stabilization of +5 °C compared to unmodified **DU1**, an antagonistic effect of lowering *T*_m_ by −5 °C compared to **DU8** was observed.

The second regularity observed in almost all duplexes is a decrease in melting temperature by approximately the same value in the range from −4.0 to −5.5 °C, caused by the presence of a cyclen-labeled U^C^ in one of the strands. The destabilizing effect of U^C^ is additive and increases with increasing number of cyclen moieties attached to a single strand and their total number in the duplex, with Δ*T*_m_ averaging −6 °C for each U^C^ introduced. However, there are two exceptions to this regularity; one of which is **DU28**, where some selectivity against mismatch TT was observed, as evidenced by Δ*T*_m_ of +1 °C. This result is not necessarily surprising, as previous work on Zn(II)–cyclen complexes has shown that cyclen is able to selectively recognize thymine by forming hydrogen bonds between the carbonyl oxygens of thymine and the cyclen amino groups [25,26]. The second exception is **DU19**, where two U^C^ units are adjacent on complementary strands of the duplex, however, their destabilizing effect is not additive. In this case, only a thermal destabilization of −5 °C was observed, corresponding to the introduction of one U^C^ unit instead of two. The ability of covalently coupled polyamines to thermally destabilize short duplexes has been reported and is discussed below. For mixed duplexes, consisting of one strand with a cyclen-modified U^C^ and the other strand with an intercalator attached, additive effects affecting the thermal stability were also observed.

## 4. Discussion

**Figure 4:**
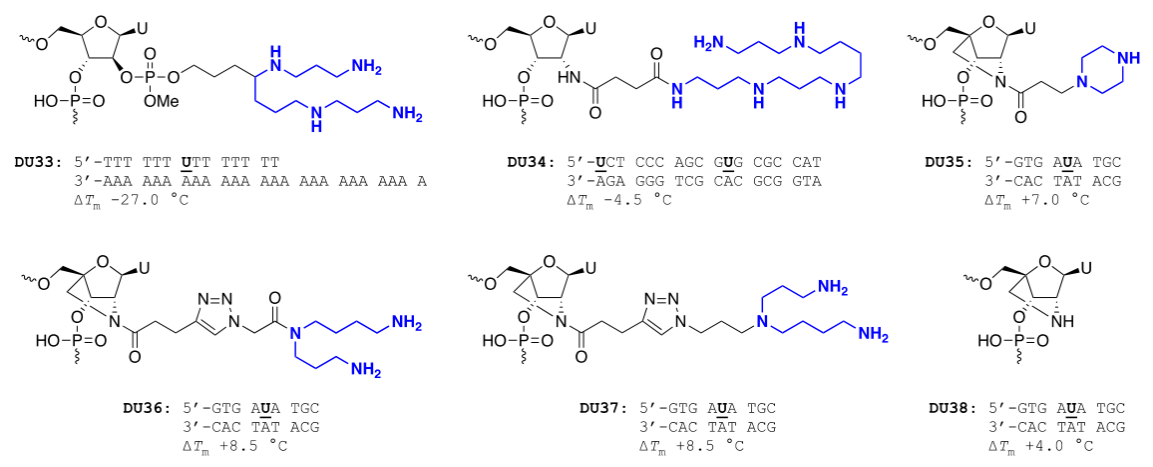
Structures and melting temperatures of polyamine–oligonucleotide conjugates known from literature. All conjugates shown are modified at the 2′-position of the ribose and are located on internal strand positions. The attached polyamines are marked in blue.

Typically, when linear polyamines are introduced at the 2′ or 4′-position of the ribose ring, either an adverse or no effect on the thermal stability of the DNA duplex is observed. Sund et al. showed that the introduction of C-branched spermine via ara-U-2′-phosphate (six-atom linker; the length of the linker is counted from the first atom attached to the ribose ring to the first polyamine atom) in the middle of **DU33** (Figure 4) decreases the thermal stability of the duplex by as much as −28.5 °C, but the same study also showed that if spermine is attached to the 3′ or 5′-end, then the thermal stability increases by +0.5 to +2.5 °C [27]. Winkler et al. attached linear polyamines via a 2′-*N*-succinylamido linker (four atoms long) to the internal and terminal nucleotides of **DU34** (Figure 4), which also reduced the thermal stability by −4.5 °C [28]. Moreover, the introduction of further polyamine moieties into the internal positions of the 18-mer only led to further destabilization of the resulting duplexes. In contrast, Wengel’s group has reported many examples of DNA oligonucleotides modified with linear or branched polyamines attached through 2′-amino-LNA motifs, including **DU35**–**DU37** (Figure 4), which increase the thermal stability from +7.0 to +8.5 °C when present in the middle of the strand [23,29,30]. The modifications present in **DU36** and **DU37** are of particular interest to us because of their sequential and structural similarity to the duplexes obtained in this work, in particular to **ON13**. The cyclen-labeled U^C^ present in **DU4** consists of three protonable amine groups, similar to the spermidine moiety in **DU37**, and a 2′-methoxy-triazolyl-butyl linker is the same length (nine atoms long) as the linkers in **DU36** and **DU37**. Despite these similarities, **DU4** shows reduced thermal stability by −4.0 °C, while **DU36** and **DU37** show increased stabilization by +8.5 °C. The stabilization effect of +7.0 °C is also maintained by a shorter propanamide linker (three atoms long) conjugated with the piperazine ring in **DU35**, and even by the mere presence of the 2′-amino-LNA motif in **D38**, resulting in Δ*T*_m_ of +4.0 °C. The results discussed here may suggest that the 2′-amino-LNA motif helps to adopt the correct conformation of the ribose ring, which may be critical for duplex stabilization by polyamines, especially when they are covalently attached to the internal positions of the strands.

The results obtained for **DU4** with those from the literature indicated a lower destabilization effect of cyclen in comparison with reported data for linear polyamines. The exceptions are polyamine–LNA conjugates, such as **DU36** and **DU37**, unambiguously confirming the stabilizing effect of the LNA motif. Our study shows that the incorporation of cyclen-labeled U^C^ in the middle of **DU4** and other related duplexes led to a decrease in the thermal stability by an average value of −6.0 °C. An interesting exception to this rule is the introduction of two U^C^ units directly opposite to each other on complementary strands, which causes much less duplex destabilization than would appear from the number of polyamines attached. Such an arrangement can be used to maximize the number of polyamines introduced with the least effect on the thermal stability of the duplex. Another exception to this rule is the presence of U^C^ opposite the TT mismatch on a complementary strand; in this case, we observed a slight stabilization of the duplex, which can be used to design mismatch-selective DNA binders as useful models for understanding and modulating the action of DNA repair enzymes. We also showed that the presence of U^T^ and U^N^ modifications has a strong thermostabilizing effect on duplex formation, and the proximity of both modifications to each other and U^C^ does not disturb their interaction with the duplex. This property can be useful to overcome the thermodestabilizing effect of cyclen moiety and to design hybrids possessing two functionalities. Shedding more light on the source of the observed effects will require additional studies on the interaction of polyamines with duplexes but will provide valuable insight into the key design requirements for such conjugates and their future applications in biological systems.

## 5. Conclusions

We have developed a new protocol for the synthesis of cyclen-containing oligonucleotides by post-synthetic coupling of azide-functionalized labels to 2′-*O*-propargylated oligonucleotides using Cu(I)-catalyzed azide–alkyne cycloaddition. All dye-containing oligonucleotides have a positive effect on the thermal stability of the obtained duplexes, especially those containing mismatches on one of the strands. The *T*_m_ amplitude depends on the number and position of the attached dye molecules. The presence of the cyclen moiety in one of the strands decreases the melting temperature by approximately the same value in the range from −4.0 to −5.5 °C. This destabilization effect can, however, be diminished by the presence of a dye molecule in the complementary strand. Compensating for the destabilizing effect of cyclen (and possibly other polyamines) on dsDNA by inclusion of an intercalating dye is a promising tool for adjusting the thermal stability of polyamine-labeled DNA duplexes.

## Figures and Tables

**Table 1:** Overall yields and MADI-TOF mass spectra of the obtained conjugates.

No	Sequence	MALDI-TOF MS		Yield	IE-Purity
		Calcd. *m*/*z* [M+H]^+^	Found *m*/*z*		
**ON6**	5′-GTG AU^3TFA^A TGC	3379.303	3379.920		
**ON6′**	5′-GTG AU^2TFA^A TGC	3283.295	3283.192		
**ON9**	5′-GTG AU^T^A TGC	3119.595	3118.754	56%	98%
**ON10**	5′-GCA TAU^T^ CAC	3048.222	3048.840	52%	99%
**ON11**	5′-GTG AU^N^A TGC	3088.618	3088.722	58%	86%
**ON12**	5′-GCA TAU^N^ CAC	3017.618	3016.764	61%	83%
**ON13**	5′-GTG AU^C^A TGC	3091.278	3091.684	41%	98%
**ON14**	5′-GCA TAU^C^ CAC	3020.734	3020.739	40%	98%
**ON15**	5′-GU^C^G ATA U^C^GC	3428.957	3428.227	38%	95%

## Data Availability

The data presented in this study are available upon request from the corresponding author.

## Supplementary Materials

The following supporting information can be downloaded at: https://www.mdpi.com/article/10.3390/pharmaceutics14010066/s1, Figure S1. (a) Semi-preparative RP-HPLC (*t_R_* 10.489 min), (b) analytical IE-HPLC (*t_R_* 8.31 min) and (c) MALDI-MS (calcd. *m/z* [M+H]+ 3091.278) of ON13; Figure S2. Analytical IE-HPLC of ON14 (*t*_R_ 9.28 min) obtained directly from ON4, without separation of *N*-TFA protected byproducts ON7”; Figure S3. (a) Semi-preparative RP-HPLC (*t*_R_ 10.173 min), (b) MALDI-MS (calcd. *m/z* [M+H]^+^ 3020.734) of ON14 obtained by separating a mixture of ON7” byproducts and their joint deprotection and (c) analytical IE-HPLC (*t*_R_ 8.31 min) of ON14; Figure S4. (a) Semi-preparative RP-HPLC of ON8” byproducts (*t*_R_ 14.476, 14.876 and 16.694 min) containing cyclen moieties with a different degree of *N*-TFA protection. These fractions were collected and deprotected together to yield the final conjugate ON15. (b) MALDI-MS of ON15; Figure S5. (a) Semi-preparative RP-HPLC (*t*_R_ 15.906 min), (b) analytical IE-HPLC (*t*_R_ 11.42 min) and (c) MALDI-MS (calcd. *m/z* [M+H]^+^ 3119.595) of ON9; Figure S6. (a) Semi-preparative RP-HPLC (*t*_R_ 15.959 min), (b) analytical IE-HPLC (*t*_R_ 11.87 min) and (c) MALDI-MS (calcd. *m/z* [M+H]^+^ 3048.222) of ON10; Figure S7. (a) Analytical IE-HPLC (*t*_R_ 10.84 min) and (b) MALDI-MS (calcd. *m/z* [M+H]^+^ 3088.618) of ON11; Figure S8. (a) Analytical IE-HPLC (*t*_R_ 10.95 min) and (b) MALDI-MS (calcd. *m/z* [M+H]^+^ 3017.618) of ON12.

## References

[1] Sharma V.K., Sharma R.K., Singh S.K. (2014). Antisense oligonucleotides: Modifications and clinical trials. MedChemComm.

[2] Wan W.B., Seth P.P. (2016). The Medicinal Chemistry of Therapeutic Oligonucleotides. J. Med. Chem..

[3] Dubey I., Dubey L., Piletska E., Piletsky S. (2007). Metal complexes of 1, 4, 7-triazacyclononane and their oligonucleotide conju-gates as chemical nucleases. Ukr. Bioorg. Acta.

[4] Stewart K.M., McLaughlin L.W. (2004). Four-Arm Oligonucleotide Ni(II)−Cyclam-Centered Complexes as Precursors for the Generation of Supramolecular Periodic Assemblies. J. Am. Chem. Soc..

[5] Wan S.-H., Liang F., Xiong X.-Q., Yang L., Wu X.-J., Wang P., Zhou X., Wu C.-T. (2006). DNA hydrolysis promoted by 1,7-dimethyl-1,4,7,10-tetraazacyclododecane. Bioorganic Med. Chem. Lett..

[6] Wang M.-Q., Zhang J., Zhang Y., Zhang D.-W., Liu Q., Liu J.-L., Lin H.-H., Yu X.-Q. (2011). Metal-free cleavage efficiency toward DNA by a novel PNA analog-bridged macrocyclic polyamine. Bioorganic Med. Chem. Lett..

[7] Li J., Zhang J., Lu Q.-S., Yue Y., Huang Y., Zhang D.-W., Lin H.-H., Chen S.-Y., Yu X.-Q. (2009). Hydrolytic cleavage of DNA by urea-bridged macrocyclic polyamines. Eur. J. Med. Chem..

[8] Zhang Y., Wang M.-Q., Zhang J., Zhang D.-W., Lin H.-H., Yu X.-Q. (2011). Synthesis, DNA Binding, and Cleavage Studies of Novel PNA Binding Cyclen Complexes. Chem. Biodivers..

[9] Lu Q.-S., Huang Y., Li J., Zhang Z.-W., Lin H.-H., Yu X.-Q. (2009). The Effect of an Amino-Acid Bridge on Binding Affinity and Cleavage Efficiency of Pyrenyl-Macrocyclic Polyamine Conjugates toward DNA. Chem. Biodivers..

[10] Asseline U. (2006). Development and Applications of Fluorescent Oligonucleotides. Curr. Org. Chem..

[11] Bag S.S., Saito Y., Hanawa K., Kodate S., Suzuka I., Saito I. (2006). Intelligent fluorescent nucleoside in sensing cytosine base: Importance of hydrophobic nature of perylene fluorophore. Bioorganic Med. Chem. Lett..

[12] Langenegger S.M., Häner R. (2005). Remarkable Stabilization of Duplex DNA Containing an Abasic Site by Non-Nucleosidic Phenanthroline and Pyrene Building Blocks. ChemBioChem.

[13] Crockett A.O., Wittwer C.T. (2001). Fluorescein-Labeled Oligonucleotides for Real-Time PCR: Using the Inherent Quenching of Deoxyguanosine Nucleotides. Anal. Biochem..

[14] Lee H.J., Kim B.H. (2019). Detection of AAG repeats through DNA triplex-induced G-cluster formation. Chem. Commun..

[15] Podder A., Lee H.J., Kim B.H. (2021). Fluorescent Nucleic Acid Systems for Biosensors. Bull. Chem. Soc. Jpn..

[16] Lee H.J., Kim B.H. (2021). Pyrene-Modified Guanine Cluster Probes Forming DNA/RNA Hybrid Three-Way Junctions for Imaging of Intracellular MicroRNAs. ACS Appl. Bio Mater..

[17] Gouda A.S., Przypis Ł., Walczak K., Jørgensen P.T., Wengel J. (2020). Carbazole modified oligonucleotides: Synthesis, hybridization studies and fluorescence properties. Org. Biomol. Chem..

[18] Telser J., Cruickshank K.A., Schanze K.S., Netzel T.L. (1989). DNA oligomers and duplexes containing a covalently attached derivative of tris(2,2′-bipyridine)ruthenium(II): Synthesis and characterization by thermodynamic and optical spectroscopic measurements. J. Am. Chem. Soc..

[19] Telser J., Cruickshank K.A., Morrison L.E., Netzel T.L., Chan C.K. (1989). DNA duplexes covalently labeled at two sites: Synthesis and characterization by steady-state and time-resolved optical spectroscopies. J. Am. Chem. Soc..

[20] Yang W., Giandomenico C.M., Sartori M., Moore D.A. (2003). Facile N-1 protection of cyclam, cyclen and 1,4,7-triazacyclononane. Tetrahedron Lett..

[21] Wutzel H., Jarvid M., Bjuggren J.M., Johansson A., Englund V., Gubanski S., Andersson M. (2015). Thioxanthone derivatives as stabilizers against electrical breakdown in cross-linked polyethylene for high voltage cable applications. Polym. Degrad. Stab..

[22] Astakhova I.K., Wengel J. (2013). Interfacing Click Chemistry with Automated Oligonucleotide Synthesis for the Preparation of Fluorescent DNA Probes Containing Internal Xanthene and Cyanine Dyes. Chem. Eur. J..

[23] Danielsen M.B., Christensen N.J., Jørgensen P.T., Jensen K.J., Wengel J., Lou C. (2020). Polyamine-Functionalized 2′-Amino-LNA in Oligonucleotides: Facile Synthesis of New Monomers and High-Affinity Binding towards ssDNA and dsDNA. Chem. Eur. J..

[24] Singh S.K., Nielsen P., Koshkin A., Wengel J. (1998). LNA (locked nucleic acids): Synthesis and high-affinity nucleic acid recogni-tion. Chem. Commun..

[25] Zhu Z., Wang S., Wei D., Yang C. (2016). Zn2+-cyclen-based complex enable a selective detection of single-stranded thymine-rich DNA in aqueous buffer. Biosens. Bioelectron..

[26] Kimura E., Katsube N., Koike T., Shiro M., Aoki S. (2002). Effects of Bis(aromatic) Pendants on Recognition of Nucleobase Thymine by Zn2+ -1,4,7,10-tetraazacyclododecane (Zn2+ -cyclen). Supramol. Chem..

[27] Sund C., Puri N., Chattopadhyaya J. (1996). Synthesis of C-branched spermine tethered oligo-DNA and the thermal stability of the duplexes and triplexes. Tetrahedron.

[28] Winkler J., Saadat K., Díaz-Gavilán M., Urban E., Noe C.R. (2009). Oligonucleotide–polyamine conjugates: Influence of length and position of 2′-attached polyamines on duplex stability and antisense effect. Eur. J. Med. Chem..

[29] Lou C., Vester B., Wengel J. (2015). Oligonucleotides containing a piperazino-modified 2′-amino-LNA monomer exhibit very high duplex stability and remarkable nuclease resistance. Chem. Commun..

[30] Lou C., Samuelsen S.V., Christensen N.J., Vester B., Wengel J. (2017). Oligonucleotides Containing Aminated 2′-Amino-LNA Nucleotides: Synthesis and Strong Binding to Complementary DNA and RNA. Bioconjugate Chem..

