# Supplementary material for: Polyamine–Oligonucleotide Conjugates: 2′-OMe-Triazole-Linked 1,4,7,10-Tetraazacyclododecane and Intercalating Dyes and Their Effect on the Thermal Stability of DNA Duplexes
Source: Pharmaceutics. 2021 Dec 28;14(1):66. doi: 10.3390/pharmaceutics14010066 (PMC8778778; doi:10.3390/pharmaceutics14010066)
Supplement: Supplementary file 1 [file pharmaceutics-14-00066-s001.zip › pharmaceutics-1496200-supplementary.pdf]

pharmaceutics

MDPI

Supplementary Materials: Polyamine–Oligonucleotide Conjugates: 2′-*O*Me-Triazole-Linked 1,4,7,10-Tetraazacyclododecane and Intercalating Dyes and Their Effect on the Thermal Stability of DNA Duplexes

Mateusz D. Tomczyk, Mariusz Zalewski, Per T. Jørgensen, Jesper Wengel and Krzysztof Walczak

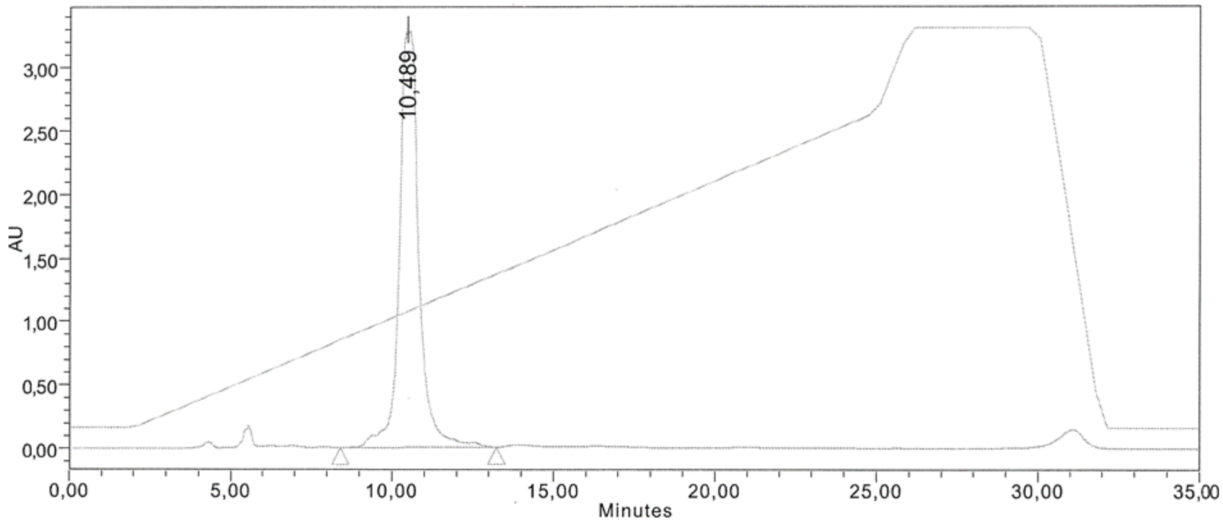

(a) RP-HPLC

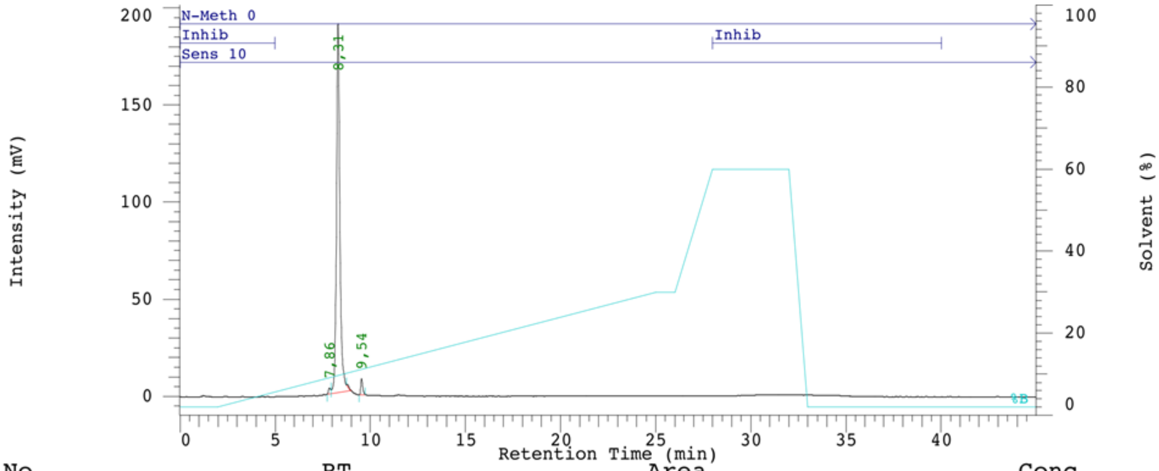

No.	RT	Area	Conc
1	7,86	20908	0,893
2	8,31	2266592	96,788
3	9,54	54317	2,319
		2341817	100,000

(b) IE-HPLC

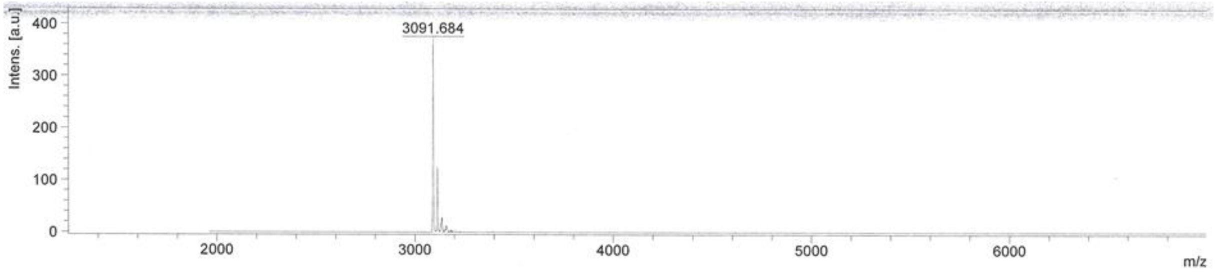

(c) MADLI-MS

Figure S1. (**a**) Semi-preparative RP-HPLC (t_R 10.489 min), (**b**) analytical IE-HPLC (t_R 8.31 min) and (**c**) MALDI-MS (calcd. *m*/*z* [M+H]+ 3091.278) of **ON13**.

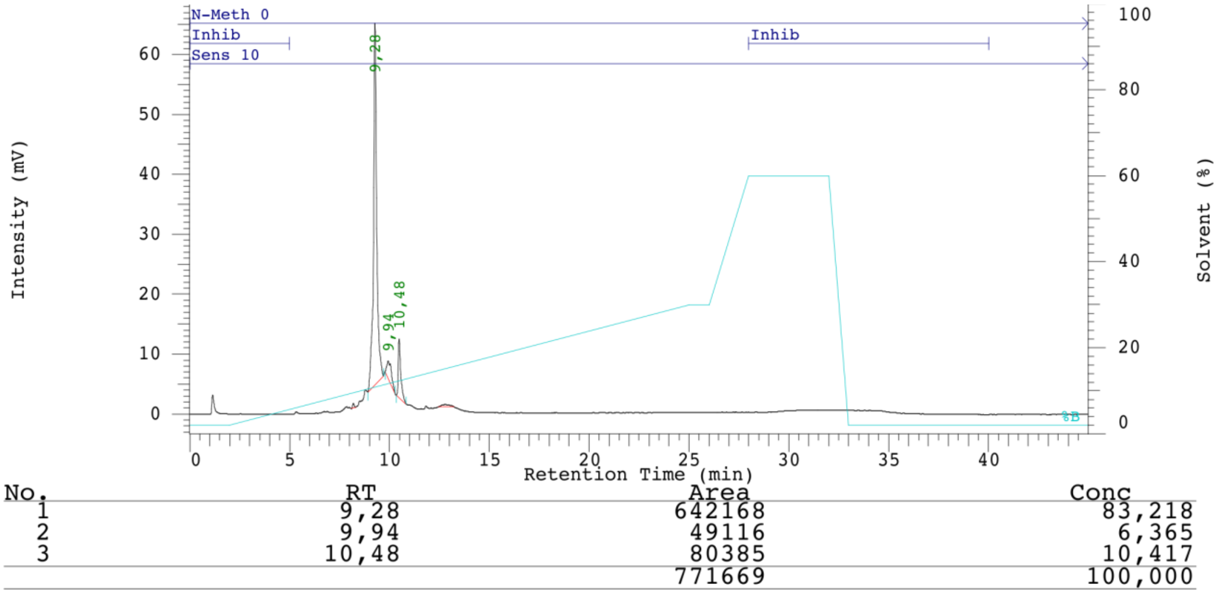

No.	RT	Area	Conc
1	9,28	642168	83,218
2	9,94	49116	6,365
3	10,48	80385	10,417
		771669	100,000

Figure S2. Analytical IE-HPLC of **ON14** (t_R 9.28 min) obtained directly from **ON4**, without separation of *N*-TFA protected byproducts **ON7"**.

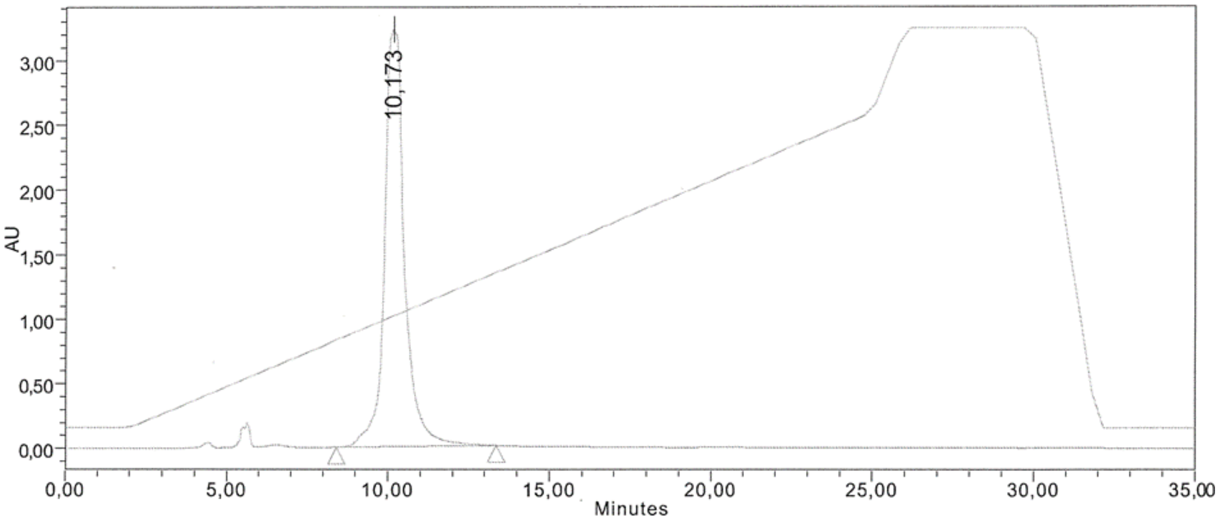

(a) RP-HPLC

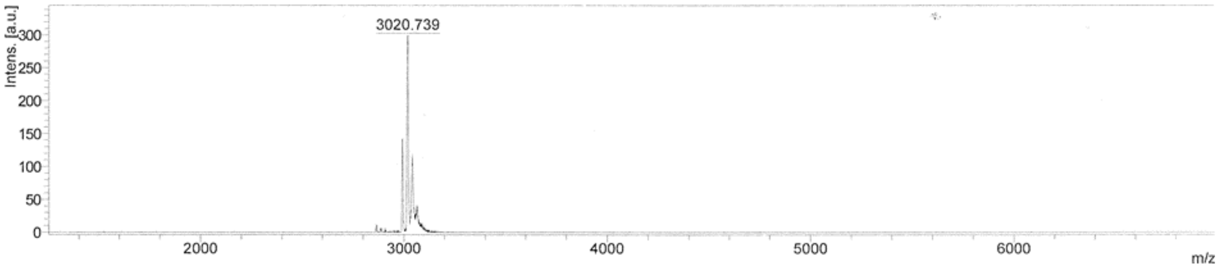

(b) MALDI-MS

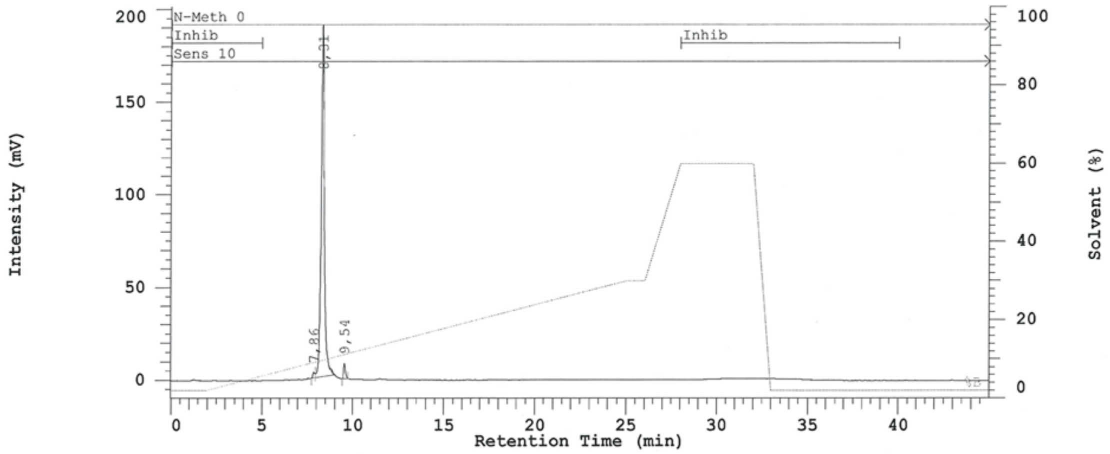

No.	RT	Area	Conc
1	7,86	20908	0,893
2	8,31	2266592	96,788
3	9,54	54317	2,319
		2341817	100,000

(c) IE-HPLC

Figure S3. (**a**) Semi-preparative RP-HPLC (t_R 10.173 min), (**b**) MALDI-MS (calcd. *m*/*z* [M+H]$^+$ 3020.734) of **ON14** obtained by separating a mixture of **ON7″** byproducts and their joint deprotection and (**c**) analytical IE-HPLC (t_R 8.31 min) of **ON14**.

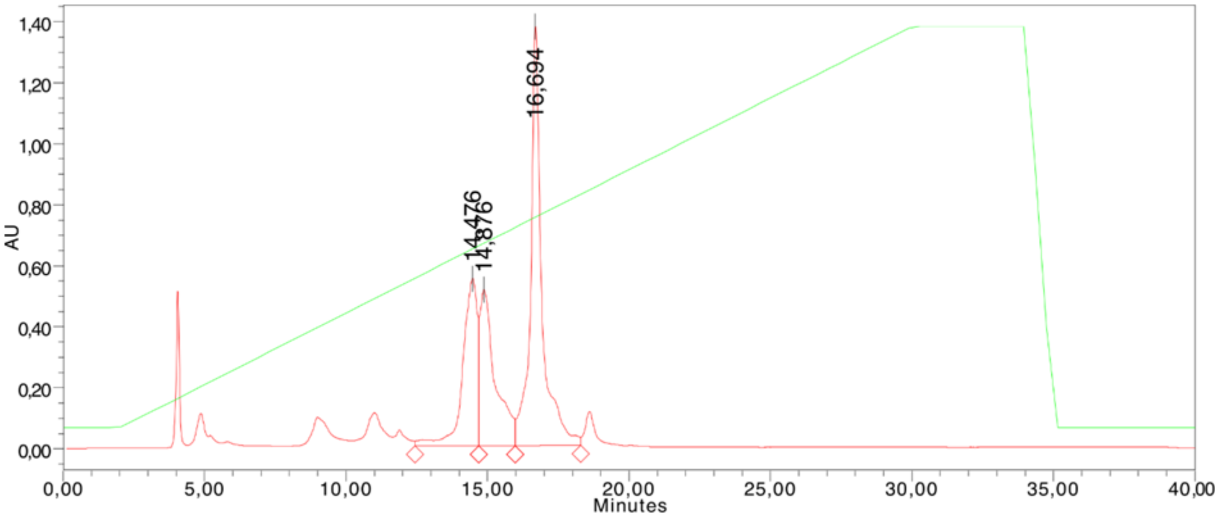

(a) RP-HPLC

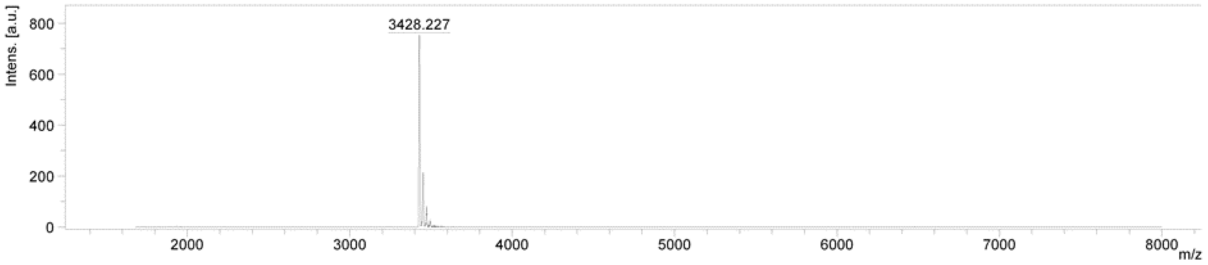

(b) MALDI-MS

Figure S4. (**a**) Semi-preparative RP-HPLC of **ON8″** byproducts (t_R 14.476, 14.876 and 16.694 min) containing cyclen moieties with a different degree of *N*-TFA protection. These fractions were collected and deprotected together to yield the final conjugate **ON15**. (**b**) MALDI-MS of **ON15**.

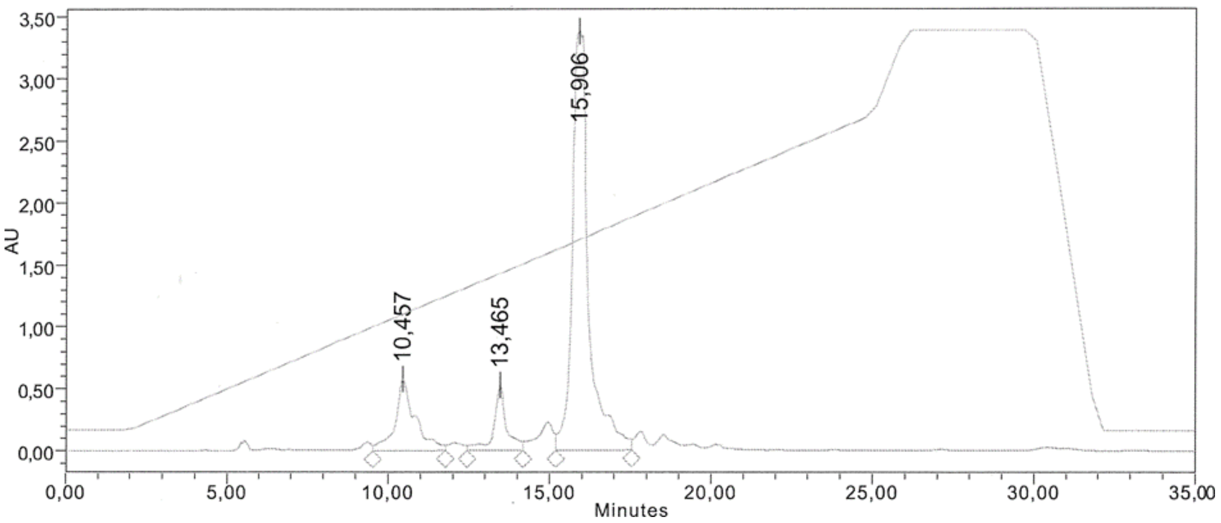

(a) RP-HPLC

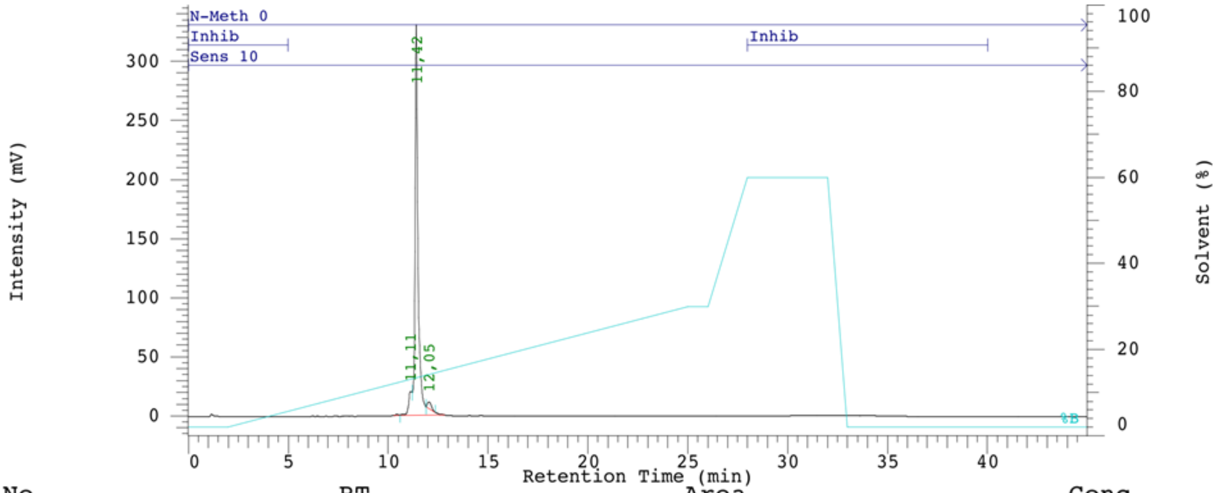

No.	RT	Area	Conc
1	11,11	252148	6,701
2	11,42	3433566	91,251
3	12,05	77070	2,048
		3762784	100,000

(b) IE-HPLC

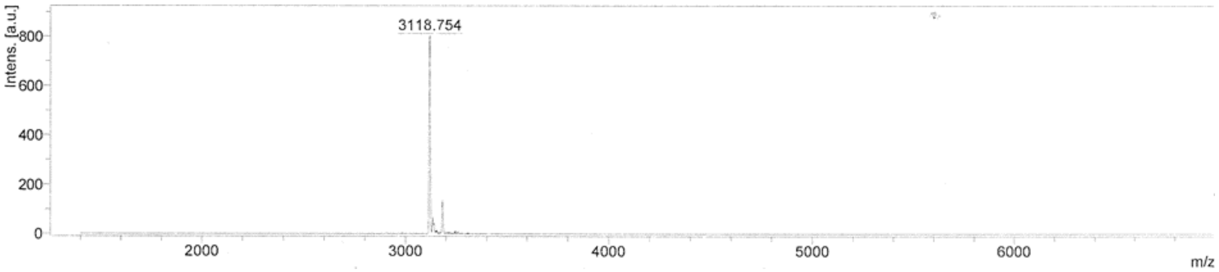

(c) MALDI-MS

Figure S5. (**a**) Semi-preparative RP-HPLC (t_R 15.906 min), (**b**) analytical IE-HPLC (t_R 11.42 min) and (**c**) MALDI-MS (calcd. *m/z* $[M+H]^+$ 3119.595) of **ON9**.

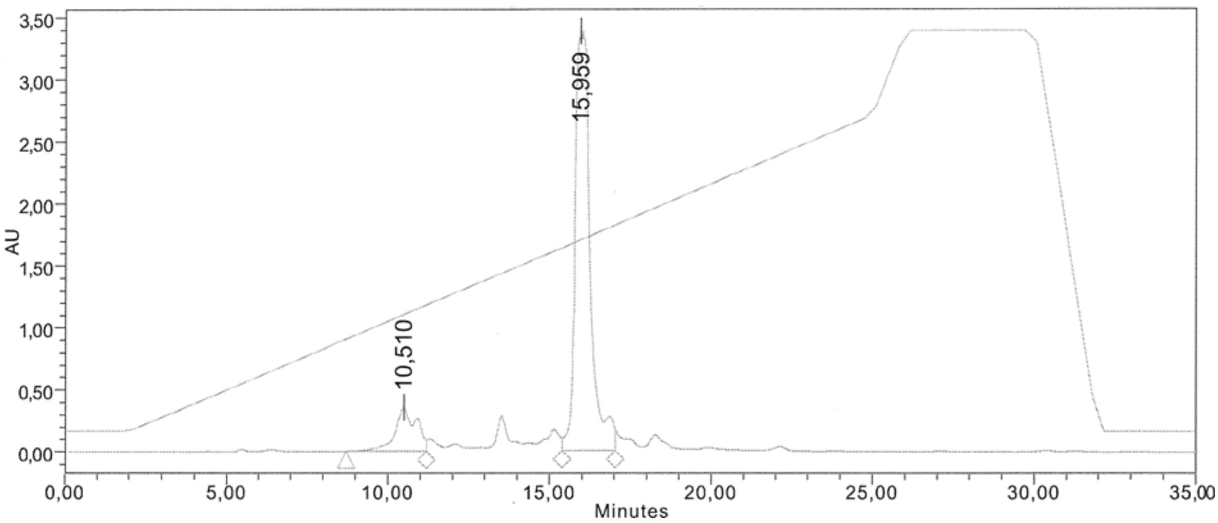

(a) RP-HPLC

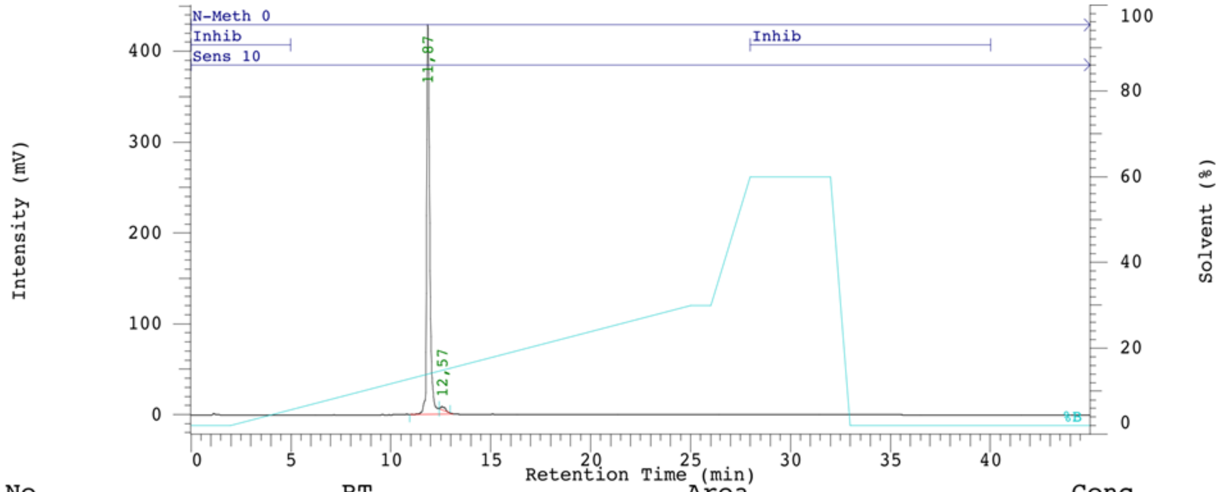

No.	RT	Area	Conc
1	11,87	4573018	98,727
2	12,57	58973	1,273
		4631991	100,000

(b) IE-HPLC

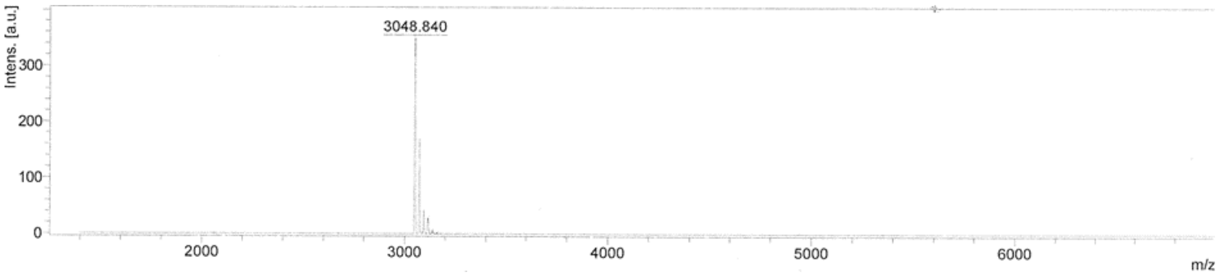

(c) MALDI-MS

Figure S6. (a) Semi-preparative RP-HPLC (tR 15.959 min), (b) analytical IE-HPLC (tR 11.87 min) and (c) MALDI-MS (calcd. m/z [M+H]+ 3048.222) of ON10.

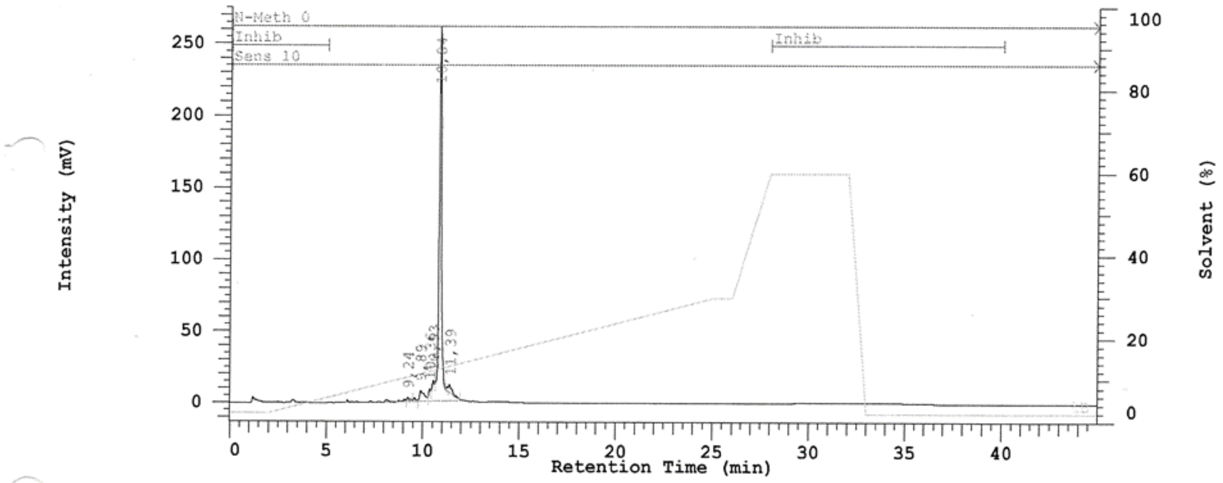

No.	RT	Area	Conc 1
1	9,24	23473	0,828
2	9,89	118065	4,167
3	10,36	58717	2,072
4	10,53	122617	4,327
5	10,84	2443785	86,244
6	11,39	66922	2,362
		2833579	100,000

(a) IE-HPLC

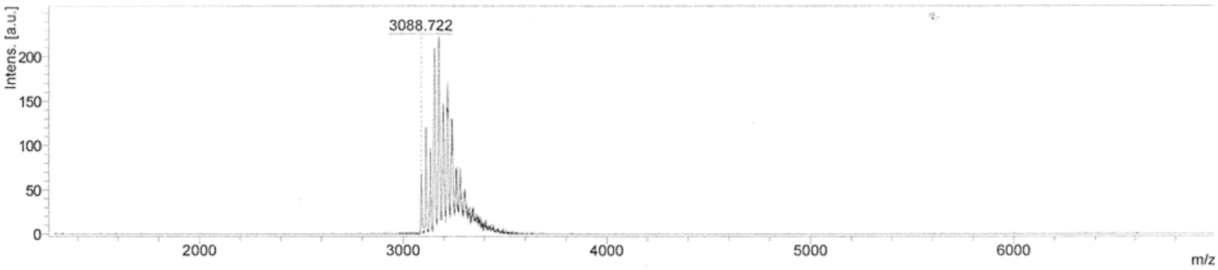

(b) MALDI-MS

Figure S7. (**a**) Analytical IE-HPLC (t_R 10.84 min) and (**b**) MALDI-MS (calcd. *m*/*z* [M+H]$^+$ 3088.618) of **ON11**.

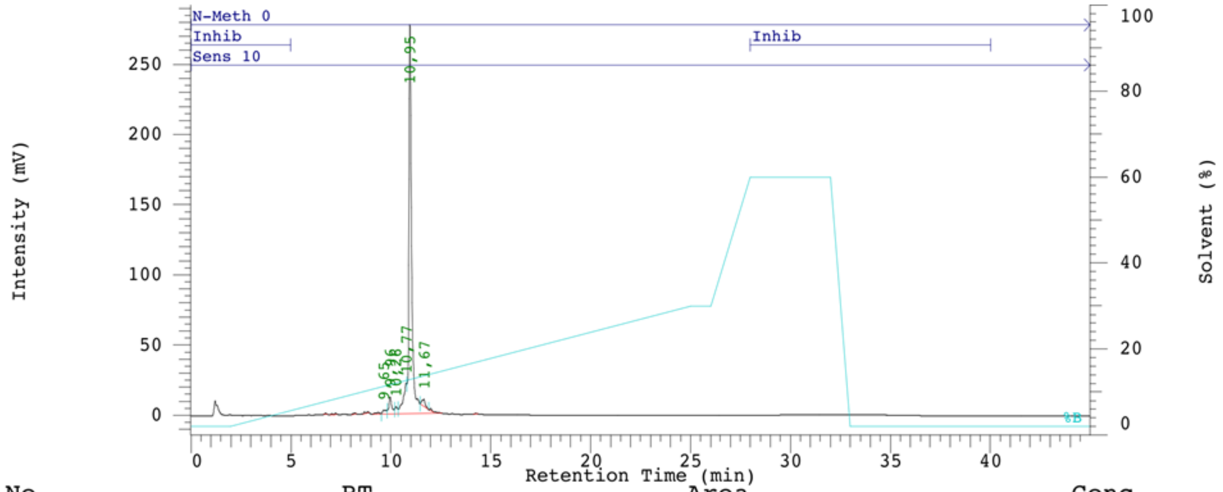

No.	RT	Area	Conc
1	9,65	35330	1,098
2	9,96	123176	3,829
3	10,26	48901	1,520
4	10,77	272676	8,475
5	10,95	2679276	83,276
6	11,67	57991	1,802
		3217350	100,000

(a) IE-HPLC

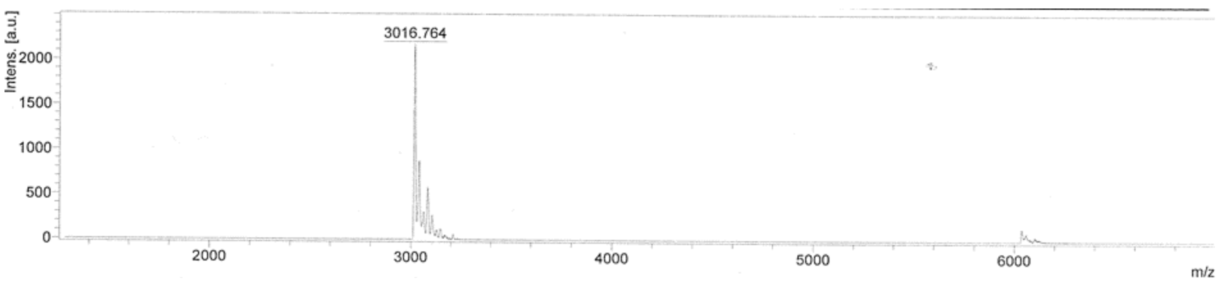

(b) MALDI-MS

Figure S8. (**a**) Analytical IE-HPLC (t_R 10.95 min) and (**b**) MALDI-MS (calcd. *m*/*z* $[M+H]^+$ 3017.618) of **ON12**.
